# Mechanisms of T cell activation: Integrating signaling pathways, experimental models, and therapeutic implications

**DOI:** 10.17179/excli2026-9572

**Published:** 2026-07-28

**Authors:** Mohammed Ageeli Hakami, Rabab Fatima, Yumna Khan, Ahad Amer Alsaiari, Jawaher Amer Alsaiari, Md Sadique Hussain

**Affiliations:** 1 Department of Clinical Laboratory Sciences, College of Applied Medical Sciences, Shaqra University, Al-Quwayiyah-19257, Riyadh, Saudi Arabia; 2 School of Health Sciences & Technology, UPES, Energy Acres, Dehradun 248007, Uttarakhand, India; 3 Saveetha Institute of Basic Medical Sciences (SIBMS), Saveetha Institute of Medical and Technical Sciences, Saveetha University, Chennai, Tamil Nadu 602105, India; 4 Department of Clinical Laboratory Science, College of Applied Medical Science, Taif University, P.O. Box 11099, Taif 21944, Saudi Arabia; 5 Uttaranchal Institute of Pharmaceutical Sciences, Uttaranchal University, Prem Nagar, Dehradun, Uttarakhand 248007, India

**Keywords:** cytokines, immunotherapy, molecular mechanisms, T cell activation, T-lymphocytes, T cell receptor signaling pathway

## Abstract

T cell activation is a central mechanism in adaptive immunity, modulating immune surveillance, host defense, and immune-mediated pathology. It is a highly coordinated mechanism including antigen recognition, co-stimulatory and co-inhibitory signaling, and cytokine-mediated regulation. This work aims to synthesize existing knowledge on the molecular mechanisms modulating T cell activation, with emphasis on integrated signaling cascades, experimental models, and therapeutic implications. A narrative review of the literature was conducted, focusing on key signaling cascades, regulatory mechanisms, and experimental methods used to analyze T cell activation in both basic and translational immunology. T cell activation is orchestrated via the mediation of T cell receptor (TCR) signaling with co-stimulatory and co-inhibitory cascades, alongside cytokine-mediated cues that collectively determine T cell fate and function. Critical intracellular signaling cascades, offer pivotal function in transcriptional and metabolic reprogramming. Experimental models such as in vitro stimulation systems, flow cytometry-based analyses, and advanced imaging approaches have significantly improved mechanistic knowledge of T cell responses. These insights have informed therapeutic approaches targeting T cell activation in cancer immunotherapy, autoimmune diseases, and infectious conditions. Although, obstacles in clinical translation remain. A comprehensive understanding of T cell activation needs integration of molecular, experimental, and clinical perspectives. Importantly, this review emphasizes the comparative strengths and limitations of contemporary experimental models and discusses how multi-omics integration and computational modeling can improve reproducibility, physiological relevance, and translational predictability in T cell research. Advancements in this field continue to offer opportunities for the development of targeted immunotherapies and enhanced disease intervention approaches.

See also the graphical abstract(Fig. 1).

## 1. Introduction

Activation of T cells is a foundation of adaptive immunity, which regulates host defense, immune tolerance and the success of immunotherapies. This process has traditionally been viewed as being initiated by a linear cascade of events undertaken by T cell receptor (TCR) interaction, but is currently considered an intensely dynamic, situation-specific process in which biochemical, biophysical, and spatial signals interact. Recent developments in high-resolution imaging, single-cell multi-omics, and mechanobiology have completely changed the way we view our knowledge about T cell decipherment of antigenic signals and their subsequent expression in a wide variety of functional effects (Courtney et al., 2018[42]). Such lessons are especially applicable in the age of cancer immunotherapy and precision medicine where even minor differences in T cell activation condition may spell out therapeutic success or failure (Nouri et al., 2021[188]).

The T lymphocytes are at the center stage of adaptive immunity, coordinating infection, neoplasia and self-antigen responses, with extraordinary specificity and sensitivity mechanisms. The core of these responses is the mechanism that involves the activation of T cells, a cascade of biochemical reactions that alters a naive or memory T cell into an effector sufficient to include cytokine secretion, cellular cytotoxicity, or immunosuppression. Though decades of research have delivered a conceptual background of T cell activation, an accumulating body of literature is fundamentally questioning the sufficiency of traditional experimental paradigms and requiring a more multi-dimensional concept of the process. It was the biochemical analysis of T cell activation that started earnestly with the generation of polyclonal pharmacological stimuli that allowed the experimental manipulation of signaling cascades in the absence of antigen specificity (Cronin and Penninger, 2007[43]). A mixture of phorbol 12-myristate 13-acetate (PMA) and ionomycin calcium ionophore turned into one of the most popular instruments in this quest. PMA activates protein kinase C (PKC) and thus nuclear factor-κB (NF-KB) whereas ionomycin participates in the increase in intracellular calcium levels which in turn activates nuclear factor of activated T cells (NFAT) transcriptional programme. By completely circumventing upstream receptor-proximal signaling, PMA/ionomycin provided both a method to induce strong cytokine production and transcriptional activation in a receptor-independent fashion, and had a valuable use in both characterising downstream effector responses and a positive control in functional assays. Coupled with pharmacological stimulation, antibody-mediated crosslinking of surface receptors came out the same strategy that was to more accurately recapitulate physiological TCR engagement. Unlike the PMA/ionomycin technique, agonistic anti-CD3 and anti-CD28 antibodies stimulate T cells via the proximal kinase ZAP-70, which phosphorylates the linker of activation of T cells (LAT), and the SH2-domain containing leukocyte protein of 76 kDa (SLP-76), thus, inducing the canonical TCR signaling pathway (Lee et al., 2023[133]). The introduction of anti-CD28 co-stimulation was especially significant, with the CD28 interaction enhancing phospholipase C-γ (PLC-γ) activity leading to the production of second messenger's diacylglycerol (DAG) and inositol trisphosphate (IP3) to initiate the full activation of T cells. Combined, these two strategies, PMA/ionomycin and anti-CD3/CD28 crosslinking, constituted the experimental basis of T cell activation studies over a number of decades, enabling the description of key signaling nodes such as PI3K6AKT, MAPK/ERK and calcineurin NFAT signaling pathways, and supporting the definition of T cell subsets and cytokine programs. Even though these stimulation platforms cannot be dismissed as useless, their reductionist approach has severe restrictions that become increasingly evident when subjected to the scrutiny of the modern world. One of the underlying issues is that neither PMA/ionomycin nor anti-CD3/CD28 antibody crosslinking reforms the spatial structure of physiological antigen recognition. T cell activation in the intact immune system takes place at a dedicated contact site between the T cell and a professional antigen-presenting cell (APC), a structure that is regulated by specific supra-molecular assembly, membrane topology and mechanobiological forces. The two classical stimulation systems activate the whole T cell surface at the same time, which prevented directionality and compartmentalization of physiological receptor interactions.

In addition to lack of spatial fidelity, pharmacological and antibody-based stimuli also produce significantly divergent transcriptional and functional effects. Single-cell RNA sequencing has provided shocking insights into transcriptomic differences in cytokine production and receptor repertoire between PMA/ionomycin-stimulated and αCD3/αCD28-activated primary human T cells, and identified activated CD4 T cell subsets that are highly induced by PMA/ionomycin activation and not mimicked by receptor-mediated stimulation (Cronin and Penninger, 2007[43]). Simulation method has a large effect at the cytokine level, with the induction of increased levels of IFN-7 and especially IL-17 under the influence of PMA/ionomycin and preferential stimulation of large amounts of IL-10 under the influence of anti-CD3/anti-CD28, culminating in a biphasic bell-shaped dose-response curve with IL-10 production under higher concentrations of PMA/ionomycin (Olsen and Sollid, 2013[189]). These observations reveal the realization that a stimulation method selection is not just a technical issue, but it directly determines a functional phenotype to a particular T cell population.

To understand T cell activation in physiological context, we should go beyond the isolated T cell and realize that the APC-T cell interface is the key component of T cell activation. Naive T cells in secondary lymphoid organs are subjected to antigen surveillance, in which they sample the surface of dendritic cells (DCs) to determine the presence of cognate peptide-MHC (pMHC) complexes. When TCR interacts, a more specialized and stable contact zone between the two cells is formed to coordinate the three different signals: antigen-specific TCR stimulation by pMHC recognition (signal 1), costimulatory receptor interactions, mainly CD28-CD80/86 (signal 2), and cytokine dependent polarization signals (signal 3) (Mariuzza et al., 2020[170]). More importantly, this is not merely a passive, docking interaction, but is a working, two-way interaction. The T cells and APCs (also including dendritic cells) have been initially perceived as being involved in a one-way information cascade where APC educates the T cell; it has become apparent that both T cell and APC cells can crosstalk in both directions at the immune synapse and that T cells can regulate APC functions (Martín-Cófreces et al., 2026[171]). This bi-directional communication involves the epigenetic and transcriptomic restructuring of DCs during synaptic confrontation with T cells, which modulators APC devolution of direction and successive presentation of antigens.

The immunological synapse (IS) is the structural equivalent of the APC-T cell interaction, and this supramolecular assembly is highly ordered and was first formally described in the late 1990s, but later detailed in significant molecular terms (Chao et al., 2025[30]; Martín-Cófreces et al., 2026[171]). IS assembly occurs upon interaction of the TCR with the particular peptide antigen being displayed in the complexed state with the MHC by the APC (Alarcón et al., 2011[4]), and is regulated by the spatiotemporal redistribution of TCR, integrins, co-stimulatory receptors, and signal transducers which permits the fine-tuning and combination of the stimuli that result in T cell causes (Finetti and Baldari, 2018[65]). The resulting redistribution gives rise to a typical bulls-eye architecture at the mature IS, with TCRCD3 complexes, and PKC levels concentrated in the central supramolecular activation cluster (cSMAC), and adhesion molecules LFA-1 and ICAM-1 concentrating at the peripheral supramolecular architecture (pSMAC), and a more distant area (dSMAC) with filamentous actin and early signaling microclusters.

The IS is not just a passive scaffold upon which an amplification of the signal is performed; it is an active platform on T cell activation magnitude and duration. The immunological synapse is the basic machinery of architecture of direct interactions and secretory crosstalk between immune cells and is believed to be a central underlying mechanism of immune evasion or inflammation found in many diseases, including tumors and infections (Leunig et al., 2025[137]). The accurate spatiotemporal organization of the IS defines the threshold in T cell activation, the polarized release of the cytokines and cytotoxic granules, and the degree of costimulatory to inhibitory signal integration. More recent studies have also shown that the formation of IS is a self-inhibitory phenomenon: ectocytosis causes TCR signaling to self-inhibitate at the immune synapse, and has offered a pathway through which long-lasting activation is attenuated (Nakamura et al., 2025[184]). A better-understood aspect of physiological T cell activation, which has been garnered growing importance, is the role of biophysical parameters that are completely inaccessible to either pharmacological or antibody-based stimulation platforms. Topping this list is the fact that mechanical force is involved in TCR triggering. Recent developments have also shown that the TCR itself is a mechanoreceptor, and a model TCR Bending Mechanosignal has been suggested in which the local bending of the T cell membrane at the nanometer scale allows sustained contact between relatively small pMHC-TCR complexes between large surface receptors and adhesion molecules on the opposing surfaces of the T cells and APCs, enhancing access of TCR signaling domains to phosphorylation (Al-Aghbar et al., 2022[3]). This mechanosensory role of the TCR is actively determined by the process of catch bond formation: the TCR establishes noncovalent catch bonds to agonist ligands in the presence of force that prolongs the lifetime of the bond and shortens the lifetime of the non-agonist ligands (the slip bonds) in the presence of force (Wu and Ye, 2025[266]). This force-sensitivity ratio thus represents a biophysical proofreading scheme that can be used to discriminate agonist and antagonist ligands based on bond kinetics and not necessarily on equilibrium affinity.

Mechanical forces enhance TCR mechanotransduction by three convergent signals; TCRpMHC binding, CD28 co-stimulation, and IL-2 receptor signaling, and epigenetic regulatory mechanisms also take part in downstream mechanotransduction to control T cell activation and performance (Jeffreys et al., 2024[111]). It revolves around the actomyosin cytoskeleton that via its binding to actin forms and unfolds the actin filaments, producing all necessary internal forces ensuring that the optimal tension exists between TCR and pMHC on the cell surface. At the same time, the first molecular events that initiate immunoreceptor signaling in the case of ligand binding have not yet been fully comprehended, and there are central issues of controversy on the TCRpMHC catch bond mechanism, the ability of T cells to generate endogenous forces, and how these forces control receptorligand kinetics (Travaglino et al., 2026[244]). In addition to the mechanical forces, the concentration and spacing of pMHC ligands on the APC surface have significant effects on the outcome of the activation. Heavy TCR signals on hard surfaces by high-density ligands result in the high mechanical force on the activating cell, which causes extensive cell spreading and high T cell activation whereas weak TCR signals on soft surfaces result in minimal activation (Hyun et al., 2023[104]). This correlation between ligand density, substrate mechanics and signaling output shows that the T cell activation thresholds are joint properties of biochemical and biophysical population parameters at the cell-cell interface, which are not inherently present in stimulation paradigms in vitro.

Although much has been known regarding the molecular components of T cell activation, there are still a number of basic questions that are yet to be answered and that constitute the leading edge of modern research work. These queries are not only intellectual intrigues but doctrines on life that would be obstacles to transformation of mechanistic understandings into long-term intercessory measures. The immune synapse is not a fixed structure but an evolving, dynamic interface in terms of its molecular composition which varies on timescales between seconds (microcluster assembly/disassembly) and hours (transcriptional reprogramming/epigenetic remodeling). It is not well understood how the T cell can then use these temporally and spatially heterogeneous inputs to form a coherent, digital decision to activate, or a graded, analogue response. The correlation between early proximal dynamics (calcium signal, ZAP-70 phosphorylation) and late transcriptional ones (I.L. 2 production, clonal expansion) will not be linear; signal integration control points and folds will lie between receptor binding indicated by the signal and gene expression indicated by the signal. According to the nominally uniform stimulation of antigen, T cells in a clonal population respond in a number of quantitatively and qualitatively different ways. This heterogeneity which occurs at the cytokine secretion level, proliferative capacity, exhaustion potential, and differentiation destiny levels has deep consequences on immunological memory, tumor immunity and the results of adoptive cell therapies.

A central aim of this review is to critically analyse the experimental systems employed to study T cell activation and to determine their suitability for mimicking physiological immune responses, in addition to the general description of signaling pathways. There are several reviews that thoroughly describe TCR signaling networks and immunological synapse biology, but fewer reviews have systematically compared the various experimental models that have been used to support these findings and their respective merits, drawbacks, and applicability for translation (Schäfer et al., 2024[214]; Yang and Poholek, 2024[272]). With the forwarding importance of reproducibility, systems immunology, and clinical translation, the choice of representation and experimental platform have become as crucial as the knowledge of the signaling pathways themselves. In this context, this review addresses both methodological modalities and mechanistic immunology, focusing on strategies for utilizing multi-omics approaches, new imaging systems, biomimetic surroundings, and mathematical models of T cell activation to make studies more physiologically relevant and predictive.

All the questions above lead to one general imperative: the field needs a conceptual and methodological shift in which the study of T cell activation has ceased being a discrete molecular event of interest and is instead approached as a multidimensional and heterogeneous process. The newest technologies such as single-cell multi-omics, cryo-electron microscopy, CRISPR-based functional screens, advanced live-cell imaging, and physiologically calibrated biomaterial platforms are all serving to redefine the concept of what T cell activation is. This review summarizes these developments in the aspect of signaling pathway architecture, experimental model systems and therapeutic implications and intends to offer a more detailed picture of signaling pathway activation in all its biological complexity of T cell activation.

## 2. Signal Initiation at the Immune Synapse

Immunological synapse (IS) is the fundamental structural basis of direct contacts and secretory crosstalk involving immune cells and crosstalk between immune cells and other cells. It is considered to be a primary causative agent of inflammation or immune evasion observed in cancer and infections, because of its dysregulation (Davis and Dustin, 2004[46]). IS is a highly structured dynamic membrane interface between the T cell and the antigen presenting cell (APC). The IS is not a passive binding system, rather it is an active signaling machine, the spatial structure of which is linked to the qualitative and quantitative product of T cell activation (Lee et al., 2002[135]; Chao et al., 2025[30]). Recent technology in live-cell super-resolution microscopy, assisted lipid bilayer reconstitution systems and single-molecule force spectroscopy have defined receptor interactions, microcluster cellularization, co-receptor regulation, and mechanosensory responses for T cell fate assessment.

### 2.1 TCR-pMHC interaction and early triggering events

T cell activation requires the presence of T cell receptor (TCR)-cognate peptide-major histocompatibility complex (pMHC) complex on the surface of the APC (Beddoe et al., 2009[15]). Apparently, it is just a simple bimolecular interaction that is regulated by a complex set of kinetic, spatial, and mechanical parameters that together define the signal fidelity and antigen discrimination.

The kinetic proofreading (KPR) model explains a famous phenomenon, according to which when a ligand first interacts with a receptor, the effective signal is not generated immediately. Rather, the phosphorylation of the tyrosine residues is done through a series of intermediate steps. This process is normally triggered by the formation of a complex between a ligand and a receptor. It means that it is the pMHC that binds to a TCR to be a complex, followed by a series of other events to the final activation. Kinetic proofreading model The theory suggests that TCR-proximal signaling occurs via cascade of reversible biochemical changes, and, most importantly, in a gradual phosphorylation of ITAM on CD3 subunits (Moffett et al., 2025[178]). A minimum dwell time is required to facilitate each modification step since the TCR-pMHC bond needs to remain intact. Weak agonists or self-peptides ligands with short half-lives dissociate before they can go through the modification cascade leading to incomplete or abortive signaling (Jansson, 2011[109]). Moreover, agonist pMHC complexes allows complete transit over the proofreading ladder and downstream signal by their extended bond lifetime (Shi et al., 2024[224]). Another well-known model known as serial engagement, conveys that a single pMHC molecule can sequentially engage and activate multiple TCR complexes before being downregulated. This catalytic mode of receptor utilization amplifies the signal proportionally to the TCR-pMHC off-rate: ligands with intermediate off-rates can serially engage more TCRs per unit time than those with extremely high or low affinities (Valitutti, 2012[247]). Serial engagement thus operates in concert with kinetic proofreading as fast-dissociating ligands engage many TCRs but provide insufficient dwell time for productive proofreading. Also, very slow-dissociating ligands may signal productively but trigger fewer total TCR engagements per APC contact (Coombs and Goldstein, 2005[41]). Both molecules bind non-polymorphic regions of MHC class II (CD4) or MHC class I (CD8 αβ heterodimer), thereby forming a trimolecular TCR-pMHC-co-receptor complex. Crucially, the cytoplasmic tails of CD4 and CD8α constitutively associate with the Src-family kinase Lck (p56lck), which is thereby recruited in close proximity to CD3 ITAMs upon co-receptor co-engagement (Shah et al., 2021[219]). Also, it is established that the T cell co-receptor CD4 greatly enhances the capacity of TCR signaling, triggered by the peptide-bound MHC molecule. Recent single-molecule studies by Rushdi et al. (2022[210]) demonstrated that cooperative binding of TCR and CD4 to the same pMHC molecule enhances antigen sensitivity by up to tenfold, underscoring the mechanistic importance of co-receptor-MHC contacts in determining the activation threshold. Using DNA origami as a molecular ruler to titrate the distance between TCR and CD4, they demonstrate that 7-nm closeness maximizes the formation of TCR-pMHC-CD4 tri-molecular bonds with pMHC, which is consistent with a tri-crystal structure (Rushdi et al., 2022[210]). CD8's cooperative engagement additionally creates catch-bond behavior wherein mechanical force prolongs the TCR-pMHC-CD8 trimolecular complex rather than accelerating dissociation (Liu et al., 2014[151]).

Even when at rest, a significant proportion of Lck is in an active, open form ready to phosphorylate CD3 ITAMs in the presence of TCR engagement (Nika et al., 2010[187]). TCR ligation and co-receptor recruitment focus Lck to the nascent signaling site where it phosphorylates the dual tyrosine motifs of the ITAMs of CD3 chains. Moreover, the high-affinity docking sites of the tandem SH2 domains of ZAP-70 are comprised of the doubly phosphorylated z-chain ITAMs (Woessner et al., 2025[264]). Lck then phosphorylates ZAP-70 on Tyr492/493 and Tyr315 zones which neutralizes the intramolecular autoinhibition and provides complete kinase activity (Bravo et al., 2025[21]). The action of regulatory phosphatases which dephosphorylate the inhibitory Lck phosphotyrosines to keep a permissive pool of active kinase, as well as restraining runaway signaling by ITAM dephosphorylation, is also regulated (Lo et al., 2018[158]).

### 2.2 Microcluster formation and signal propagation

The receptors do not signal as dispersed monomers upon TCR engagement but instead rapidly coalesce into discrete, nanoscale assemblies. This constitutes the major functioning signaling units of IS and are known as TCR microclusters (Balagopalan et al., 2020[12]). T cell activation begins before the full immunological synapse (IS) forms. Instead, early signaling occurs in small clusters called TCR microclusters (MCs), where T cell receptors, kinases, and adaptor proteins to initiate activation. These microclusters then move toward the center of the cell interface to form the central supramolecular activation cluster (cSMAC). This movement is first driven by the actin cytoskeleton and later by dynein along microtubules, especially under strong TCR stimulation (Dustin, 2008[59]). While activation signals originate in TCR microclusters, cell adhesion is organized separately through integrins. A structure called the “microsynapse” forms, consisting of a core of TCR microclusters surrounded by an adhesion ring of integrins and focal adhesion molecules. This microsynapse plays a crucial role in supporting activation, particularly when TCR stimulation is weak, by reinforcing signaling through cell-cell adhesion. Microclusters form within seconds of pMHC contact at the distal SMAC (dSMAC) and peripheral SMAC (pSMAC). Each cluster contains tens to over one hundred TCR complexes along with co-recruited signaling proteins including Lck, ZAP-70, LAT, and SLP-76, creating a high-density microenvironment that dramatically increases the local concentration and effective collision frequency of signaling enzymes and substrates (Hashimoto-Tane and Saito, 2016[95]).

The scaffold protein LAT (Linker for Activation of T cells) is a palmitoylated transmembrane adaptor that nucleates a multimolecular signaling complex upon ZAP-70-mediated phosphorylation. In addition to plasma membrane-associated LAT, an intracellular pool resides on recycling endosomes (Balagopalan et al., 2015[11]). VAMP7-mediated recruitment of this subsynaptic vesicular LAT pool to TCR activation sites is required for full LAT-dependent signaling (Larghi et al., 2013[129]). After TCR interaction, LAT signaling clusters assemble directly at the plasma membrane, gradually forming one molecule at a time instead of pre-formed units. Tyrosine phosphorylation of LAT is necessary for this assembly. In early T cell signaling, the slow, stepwise accumulation of these clusters functions as a kinetic bottleneck, regulating the rate at which activation signals are produced. This helps in determining the fate of the T Cell and its reaction to the antigenic peptide by slowing the speed and signal propagation probability. This further aids the specificity and sensitivity of immune activation at its earliest stage (Mori et al., 2021[181]; Lou et al., 2025[160]).

T cell development and functioning require the precise remodeling of the actin cytoskeleton that is crucial in cell division, migration, immunological synapse, and signal transduction. Examples of actin regulators are nucleators and binding proteins that mediate cytoskeletal dynamics to ensure efficient antigen recognition and signaling. Furthermore, the extracellular cues are linked to actin rearrangements which affect the conventional T cell activation and function (Lam and Chong, 2025[127]).

The control of the actin cytoskeleton in T cells is particularly significant to understand because actin dynamics dysregulation is the major cause of immunodeficiencies and autoimmunity. Actin cytoskeleton is not only a passive framework but also an active organizer and transducer of TCR signaling. In this context, further engagement of TCR leads to Vav1-mediated activation of Rac1 and Cdc42 which causes branched actin polymerization at the cell periphery to form the retrograde centripetal flow that carries TCR microclusters to the cSMAC site (Gómez-Morón et al., 2025[82]).

Instead of initiating in the mature synapse, signaling occurs in small yet dynamic TCR microclusters recruiting downstream molecules and initiating early activation. The costimulatory signaling is also incorporated in the same sites where CD28 establishes cooperative microclusters with the TCRs to enhance the original signals. With maturation of the synapse, the CD28 further rearranges into separate sections and goes on to signal by itself. This spatiotemporal organization of TCR and CD28 microclusters is the key to efficient activation and T cell's response fine-tuning (Yokosuka and Saito, 2009[277]). Another well-known model is poroelastic model which conceptualizes that the cortical actin network bestows a porous medium with a mesh size that dictates the diffusional accessibility of the signaling proteins primarily to TCR microclusters. The small size of the pore in the pSMAC effectively links signaling complexes to actin flow whereas the larger pores of the dSMAC allows finer protein recruitment in this model (Yokosuka and Saito, 2009[277]; Yuan et al., 2018[280]).

### 2.3 Co-stimulatory and co-inhibitory modulation

The co-stimulatory and co-inhibitory modulation in the immune system is known well to be associated with Type 2 Diabetes. TCR-derived Signal 1 in combination with co-stimulatory signal 2 on the surface of APC has to be integrated to yield productive T cell activation. On the other hand, co-inhibitory receptors generate molecular brakes that prevent hyperactivation of the immune system and self-tolerance (Edner et al., 2020[61]). The prototype co-stimulatory receptor, CD28, interacts with B units of APCs and sends a qualitatively new signal that potentiates but does not replace TCR signaling. Upon engagement, CD28's cytoplasmic motif is phosphorylated and recruits the regulatory subunit of PI3 kinase (PI3K), generating PIP3 and activating PDK1-Akt signaling (Acuto and Michel, 2003[1]). Importantly, CD28 co-stimulation augments LAT signalosome function by promoting lipid raft availability and increasing the phospho-LAT accessibility, furthering a positive feedback loop on TCR-proximal signaling pathway (Michel et al., 2001[177]).

The inducible co-stimulator ICOS (CD278), a CD28 family member expressed on activated T cells, binds ICOSL (B7h/B7-H2) and preferentially engages the p110δ isoform of PI3K, in contrast to CD28, which primarily activates p110γ/δ, thereby generating high levels of PIP3 and robustly activating Akt (Gonzalo et al., 2001[84]). 4-1BB (CD137), a TNFR superfamily member expressed on activated T cells, engages TRAF1/2 adaptor proteins upon 4-1BBL binding, activating the NF-κB (canonical and non-canonical), MAPK, and PI3K-Akt pathways. Uniquely, 4-1BB co-stimulation promotes sustained CD8+ T cell survival via Bcl-2/Bcl-xL upregulation, T cell memory formation, and reinvigoration of exhausted tumor-infiltrating lymphocytes (TILs) (Lee et al., 2002[132]). The discrete non-overlapping signaling programs of CD28, ICOS, and 4-1BB renders them therapeutically orthogonal. Each agonizes a distinct temporal and functional window of the T cell response, a consideration now actively exploited in bispecific T cell engager and CAR-T cell engineering strategies (Shilling et al., 2009[226]).

Through its immune receptor, the tyrosine-based switch motif (ITSM), programmed cell death protein 1 (PD-1), an immunological checkpoint cell membrane receptor, adversely controls T cell activation (Qi et al., 2020[198]). PD-1 is upregulated on activated T cells and delivers its inhibitory signal upon engagement of its ligands. The cytoplasmic tail of PD-1 harbors two tyrosine motifs, an ITIM (immunoreceptor tyrosine-based inhibitory motif) and an ITSM (immunoreceptor tyrosine-based switch motif) (Keir et al., 2008[120]).Upon phosphorylation, the ITSM preferentially recruits SHP-2 (Src homology 2 domain-containing phosphatase 2), and to a lesser extent SHP-1. SHP-2 also dephosphorylates the main TCR-proximal substrates such as CD28, ZAP-70 and LAT destroying the signaling scaffolds necessary to activate T cells productively. Importantly, PD-1 SHP-2 axis acts more upon the CD28 co-stimulatory pathway than upon TCR signaling itself. This is indicative of the main action of PD-1 is to counter the amplification of signaling provided by CD28 as opposed to abrogating the basal TCR signaling itself, which itself has profound therapeutic importance (Castro-Sanchez et al., 2020[26]). Moreover, CTLA-4 (CD152), constitutively expressed on regulatory T cells and transiently upregulated on activated T cells, operates primarily during the priming phase in secondary lymphoid organs by outcompeting CD28 for B7-1/B7-2 binding with approximately 20-fold higher affinity (Engelhardt et al., 2006[62]). Tumors exploit both PD-1 and CTLA-4 pathways, PD-L1 upregulation extinguishes effector function in the tumor microenvironment, while CTLA-4 activity on intratumoral regulatory T cells suppresses anti-tumor priming (Gascoigne et al., 2016[75]; Chen et al., 2020[32]; Hossen et al., 2023[98]). In order to minimize over-activation, avoid excessive negative selection during development, and regulate T-cell differentiation, especially in regulatory T cells (Tregs), T cells use signal dampening in the T cell receptor (TCR) pathway. T cells are able to discriminate between weak self-peptide-MHC connections (positive selection) and strong ones (negative selection) thanks to a process known as attenuation or negative regulation (Staton et al., 2011[236]).

### 2.4 Mechanotransduction in T cell activation

An emerging paradigm in TCR holds that it not only as a biochemical receptor but as a mechanosensor, integrating piconewton-scale forces generated at the T cell APC interface to improve antigen sensitivity and discrimination. Mechanotransduction is the process by which cells translate mechanical stimuli from their surroundings into intracellular biochemical signals. T cells, which are essential to adaptive immune responses, are a prime example of how mechanotransduction is necessary for their growth, differentiation, and operation. (Rushdi et al., 2020[209]). Advances in force-sensing technologies have enabled quantitative assessment of cellular mechanotransduction, revealing how biomechanical forces regulate receptor activation, intracellular signaling, and immune-cell function (Liu et al., 2025[156]). Similar biomechanical principles have been demonstrated in regenerative biomaterials and vascular tissue models, emphasizing the importance of mechanical cues in cell fate regulation (Han et al., 2025[93]). These effects profoundly create a change in interactions between receptors and ligands, a change in protein conformation, discrimination of antigens and also ensured specific cell death. Activation of TCR is not simply chemical, but is a process controlled by mechanical means, in which force applied increases, stabilizes, and assists in determining whether a productive immune signal is produced. Force-dependent TCR triggering the concept of mechanical forces being necessary to activate TCR, and not simply ligand binding. In cases when a T cell interacts with an antigen- presenting cell, TCR is bound to peptide-MHC (pMHC) complexes. In the course of this interaction, tensile forces are produced by the cytoskeleton of the T cell (particularly the flow of actins) and physically force the TCR-pMHC bond. These forces cause both conformational and kinetic alteration in the receptor complex that facilitates intracellular signaling (Ma and Finkel, 2010[165]). Experiments using seminal biomembrane force probe (BFP) and optical tweezer methods determined that the piconewton regime of tangential forces could be used. Downstream signaling can be directly triggered by to the TCR-pMHC bond, without receptor crosslinking (Moldovan et al., 2023[180]). Molecular tension probes have measured T cells to generate endogenous forces on TCR-pMHC bonds of the range 12-19 pN in a signal dependent manner. These forces are created due to the interaction of the microvilli dynamics at the initial scanning of APC, retrograde actin flow at the time of IS formation, and the contractile force of myosin IIA (Travaglino et al., 2026[244]).

Molecular bonds, when subjected to applied force, tend to either show slip behavior (bond lifetime decreases with applied force), or catch behavior (seasonally increases in bond lifetime with applied force up to a certain point, and then slips off at very high forces), depending on the specific bond type (Choi et al., 2023[38]). Weak ligands (partial agonists, antagonists) make slip-only bonds which are shortened by force, and do not have any advantage of proofreading. This dichotomy gives a strong physical process of antigen discrimination which works together with kinetic proofreading (Liu et al., 2014[151]). TCRb constant domain structural basis of TCR catch bonds explained a force-dependent conformational transition in the TCRb constant domain that allosterically controls pMHC binding through a partial unfolding of the Va-Ca and the MHC a1a2 joints (Mariuzza et al., 2020[170]). The 55 TCR- pMHC datasets and the prediction of T cell signaling outcomes based on the profile of bond lifetime demonstrated in a mechanistic modeling study of Choi et al. (2023[38]) that catch bond models could quantitatively classify such datasets and predict the outcome of T cell signaling (Choi et al., 2023[38]; Qin et al., 2025[199]). Due to the effect of the actin network which converts the binding of ligands into activation signals, cytoskeletal tension is critical to effective TCR-pMHC interaction. This stress causes the TCR- CD3 complex to undergo conformational changes thus leading to phosphorylation and consequent signaling. It also enhances the antigen discrimination of the ions by stabilizing strong antigen contacts by a force-dependent (catch bond) behavior, but weaker associations dissolve quickly (Colin-York et al., 2019[40]). Actin retrograde flow and myosin IIA contractility generate a centripetal inward tension that is transmitted through the TCR to the engaged pMHC, producing the shear and tangential forces necessary for catch bond formation (Yi et al., 2012[276]). This inside-out force generation creates a mechanotransduction loop. Here an initial TCR engagement triggers localized Vav1-mediated actin nucleation (via Rac1-Arp2/3); the resultant actin dynamics exert force on the nascent TCR-pMHC bond; force-extended bond lifetime increases ITAM phosphorylation; and enhanced ZAP-70/Vav1 signaling further drives actin remodeling, a positive feedback cycle that amplifies weak antigen signals and simultaneously discriminates against non-agonist ligands (Ksionda et al., 2012[123]). The question of whether TCR microclusters are the initiating drivers or the downstream consequences of signaling has been a subject of unresolved debate that cuts to the heart of IS biology. Early signaling events such as TCR phosphorylation occur at the nanoscale within pre-organized membrane “protein islands” containing TCR, Lck, and LAT. These nanoclusters can initiate signaling before visible microclusters form, and evidence from CAR T cells shows that full LAT scaffold assembly is not strictly required for initial clustering.

## 3. Downstream Signaling Networks: Integration and Decision-Making

After signal initiation at the immune synapse and the described proximal amplification processes in Section 2, four majors, parallel, and highly interconnected downstream axes, the Calcium NFAT signal transduction, the Ras-MAPK cascade, the NF-kB signal transduction, and the PI3K-Akt-mTOR signal transduction, decode the resulting biochemical signal.

### 3.1 Calcium-NFAT axis

Phospholipase C-γ1 (PLCγ1), activated downstream of the LAT-SLP-76 signalosome by the Tec-family kinase ITK (IL-2-inducible T cell kinase) in concert with ZAP-70-mediated phosphorylation at Tyr783, cleaves the plasma membrane phospholipid phosphatidylinositol-4,5-bisphosphate (PIP2) into inositol-1,4,5-trisphosphate (IP3) and diacylglycerol (DAG). IP3 binds with high affinity to its cognate receptor (IP3R) on the endoplasmic reticulum (ER) membrane, triggering rapid passive efflux of Ca^2+^ from the ER lumen into the cytoplasm (Park et al., 2020[192]). This initial release elevates cytoplasmic from a resting level of approximately 100 nM to a transient peak of 500-800 nM, sufficient to begin calcineurin activation; however, this finite ER Ca^2+^ pool is rapidly depleted, and alone is insufficient to sustain the prolonged, elevated intracellular Ca^2+^ concentrations necessary for production of nuclear factor of activated T cells (NFAT) activation and cytokine gene transcription (Belmont et al., 2017[16]). Sustained Ca^2+^ elevation therefore depends obligatorily on plasma membrane Ca^2+^ influx through store-operated calcium entry.

Store-Operated Calcium Entry (SOCE) via Ca^2+^ Release-Activated Ca^2+^ (CRAC) channels constitutes the dominant mechanism for sustained Ca^2+^ influx in T cells, and is indispensable for T cell activation, proliferation, and effector cytokine production (Smyth et al., 2010[232]). The molecular identity of CRAC channel was definitively established by genome-wide RNAi screens identifying Orai1 as the pore-forming subunit of the channel, and by the parallel discovery of STIM1 (Stromal Interaction Molecule 1) as the ER-resident Ca^2+^ sensor (Gudlur and Hogan, 2017[86]). Individual second messengers (Ca^2+,^ DAG, and PIP3) are converted into specific transcriptional and metabolic programs by each axis, and the physiological importance of this pathway is strong as loss of functionality (LOF) mutations in ORAI1 or STIM1 result in severe combined immunodeficiency (SCID), a lack of T cell proliferation, and an extreme deficiency of cytokine production in patients (Silva-Rojas et al., 2020[230]). Furthermore, Samakai et al. (2016[213]) demonstrated that full NFAT engagement requires transcriptional upregulation of STIM1 and PMCA4 through the cooperative activity of the zinc finger transcription factors EGR1 and EGR4, establishing a feedforward transcriptional loop that sustains the SOCE-NFAT axis over the hours-long timescale of a productive TCR-mediated response (Samakai et al., 2016[213]).

At elevated cytoplasmic Ca^2+^ concentrations, Ca^2+^ /calmodulin binds and activates calcineurin (protein phosphatase 2B), a Ca^2+^ /calmodulin-dependent serine phosphatase. Calcineurin dephosphorylates 13 serine residues within the N-terminal regulatory domain of NFAT family members (NFATc1-NFATc4), cooperative dephosphorylation of which (Hill coefficient estimated at n ≈ 6, derived from NFATc2 cooperativity modeling) drives a conformational switch that exposes a nuclear localization sequence (NLS), enabling rapid NFAT nuclear import with a half-time of approximately one minute as measured by multiphoton intravital microscopy (Li et al., 2004[140]). Nuclear NFAT cooperates with AP-1 (Fos-Jun) dimers on composite NFAT-AP-1 binding sites (with the consensus sequence AGGAAA-N4-TGASTCA) to activate canonical immune response genes including Il2, Ifng, and Il4 (Macián et al., 2001[166]). Critically, NFAT occupancy without AP-1 co-occupancy which occurs when Ca^2+^ signaling persists in the absence of adequate Ras-ERK activity, as in tumor-infiltrating T cells under chronic antigen stimulation with attenuated co-stimulation drives expression of anergy and tolerance genes such as Egr2, Grail (Rnf128), and Cbl-b, as well as PD-1 (Wisniewska et al., 2007[262]). A dual Erk/NFAT live-cell reported Erk and NFAT signaling dynamics which are indistinguishable across different pMHC inputs during the first nine hours of stimulation but diverge substantially thereafter. In that case long-timescale Erk dynamics encode pMHC affinity (quality), while NFAT dynamics encode pMHC dose (quantity) temporal information that is decoded by cis-regulatory enhancer elements to generate pMHC-specific transcriptional programs, including graded induction of IRF4 and Il2ra (Mognol et al., 2017[179]; Wither et al., 2023[263]).

### 3.2 MAPK pathway

The second TCR-derived second messenger, diacylglycerol (DAG), generated co-stoichiometrically with IP3 by PLCγ1, activates two functionally complementary Ras guanine nucleotide exchange factors (RasGEFs) in T cells. They are known as RasGRP1 (Ras Guanyl nucleotide Releasing Protein 1) and SOS1 (Son of Sevenless 1). RasGRP1, which is restricted to the nervous and hematopoietic systems, is activated by direct DAG binding through its C1 domain and by PKCθ mediated phosphorylation, translocating to DAG-enriched membrane microdomains where it converts Ras-GDP to Ras-GTP. SOS1, recruited constitutively to the LAT signalosome via Grb2 binding to phospho-Tyr171 on LAT, possesses both a catalytic RasGEF (Cdc25) domain and a distal allosteric pocket (on the REM domain) that binds Ras-GTP and increases SOS1 catalytic activity up to 80-fold upon occupancy (Denny et al., 1999[50]; Hsu et al., 2023[101]). RasGRP regulates Ras signaling both by directly activating Ras and by enabling activation of SOS through RasGTP binding to its allosteric site. This creates a positive feedback loop in which RasGTP enhances further SOS activity, amplifying the signal. Since SOS depends on RasGTP for its own activation, RasGRP1 is essential for initiating this process. In its absence, the feedback loop cannot start efficiently, though introducing active Ras can bypass this requirement and stimulate SOS. The coordinated action of RasGRP and SOS produces a highly sensitive and robust signaling response, allowing lymphocytes to respond effectively even to low levels of stimulation (Roose et al., 2007[207]).

Das et al. (2009[45]) showed single-cell flow cytometric measurement of phospho-ERK in Jurkat T cells and a stochastic simulation framework, using quantitative methods. They infer that at moderate doses of antigens, the activation of ERK is bimodal: cells either reach maximal pERK levels or maintain near-basal levels and that the proportion of digitally activated cells increases with increasing graded antigen dose, but not the amplitude of activation per cell. This positive feedback loop SOS1-PRR is blocked by RasGRP1 generated Ras-GTP which occupies the allosteric pocket of SOS1, increasing catalytic rate of SOS1 by dramatic margins to promote cooperative bistable Ras activation. More importantly, in SOS1 genetically ablated cells, the activation of ERK occurs in a purely analog and proportional and graded manner, which proves that the positive feedback loop via SOS1 allosteric pocket is the mechanistic origin of digital ERK response (Das et al., 2009[45]). Moreover, Ras activation is hysterical (memorizing). This is set off above threshold, cells retain high Ras-GTP even at stimulation levels that are below the activation threshold a behavior which could help in commitment to the activated state after antigen encounter (Chakraborty et al., 2009[28]).

### 3.3 NF-κB signaling

NF-kB signaling is one of the regulatory factors that convert the strength of TCR signal to graded gene expression in T cells. The binding of the TCR with the peptide MHC is determined by the strength and duration of T cell responses, which drive cell division, cell expansion and expression of activation related proteins. Although certain signaling pathways downstream of the TCR are activated in an all-or-none (digital) manner, others such as NF-kB are graded in signal-strength-dependent manner (Gallagher et al., 2021[70]). TCR/CD28 co-stimulation of the immune synapse selectively recruits protein kinase C-theta (PKCth), the main PKC isoform in T cells, to the central supramolecular activation cluster (cSMAC) of IS.

Protein kinase C-theta (PKCθ), the dominant PKC isoform in T cells, is selectively recruited to the central supramolecular activation cluster (cSMAC) of the immune synapse upon TCR/CD28 co-stimulation. This is primarily mediated through its C1 and C2 domains binding DAG at the inner leaflet of the plasma membrane and through CD28-PYAPP motif interactions via FilaminA and RACK1. PKCθ-cSMAC recruitment distinguishes it from other PKC isoforms and is a prerequisite for productive NF-κB activation (Isakov and Altman, 2002[107]). It is also known that only IS-localized PKCθ, not cytoplasmic PKCθ, supports downstream signaling. PKCθ phosphorylates the CARMA1 (CARD11) linker domain at multiple serine residues (including Ser552 and Ser645), disrupting the intramolecular auto-inhibitory contacts between the CARD and the linker and exposing the CARD domain for downstream assembly. The GLK (MAP4K3) kinase, recruited to the TCR/LAT/SLP-76 signalosome, additionally phosphorylates PKCθ at Ser538, a modification specifically required for NF-κB induction but not for ERK or NFAT activation establishing a regulatory input into NF-κB arm of TCR signaling (Zhang et al., 2013[284]).

The costimulatory receptor, on the other hand, is a potent inducer of NF- kB activation in T cells, and it is important to note that it forms a signaling complex with its ligand. The activation of NF-kB by its assembly in membrane microdomains allows it to activate NF-kB even in the absence of continued engagement of TCR. This prolonges the signaling and plays a key role in preventing effector and memory T cell death following antigen clearance, which emphasizes the role of integrating TCR signals in the cosmostimulatory receptor in maintaining long term immune responses (So et al., 2011[233]). PKCθ-dependent multi-site serine phosphorylation of the linker induces charge-charge repulsion that unfolds CARMA1 from this closed conformation, exposing the N-terminal CARD domain. The exposed CARMA1 CARD recruits BCL10 through heterotypic CARD-CARD interactions, with CARMA1's basic patch engaging BCL10's acidic patch, an electrostatic complementarity that distinguishes this interaction from other CARD-CARD complexes where hydrophobic contacts dominate (Gaide et al., 2001[68]; Meininger and Krappmann, 2016[173]). MALT1 contributes to NF-κB activation through dual mechanisms: its scaffolding function (recruiting TRAF6 and facilitating TAK1 activation) and its paracaspase enzymatic activity, which cleaves and inactivates negative NF-κB regulators. MALT1 substrates include A20 (TNFAIP3, a K63-deubiquitinase that terminates IKK activation); CYLD (a K63-deubiquitinase targeting TRAF proteins); and Roquin-1/MCPIP1 (a post-transcriptional repressor of cytokine mRNAs) (Staal et al., 2011[235]; Zheng et al., 2017[288]).

### 3.4 PI3K-Akt-mTOR pathway

TCR signaling additionally activates PI3K through Vav1 and directly through RasGTP-mediated recruitment of PI3Kγ (p110γ) (Reynolds et al., 2002[205]). PI3K phosphorylates the 3 position of PIP2 to generate PIP3, which serves as a membrane docking site for proteins harboring pleckstrin homology (PH) domains, including both the master kinase PDK1 and the effector kinase Akt (Tybulewicz et al., 2003[246]). All hemopoietic cells contain the 95-kDa protein Vav1, which is quickly tyrosine phosphorylated after T cell antigen receptor (TCR) stimulation. A Dbl homology domain, a distinguishing feature of a guanine nucleotide exchange factor (GEF) for Rho-family GTPases, is one of the many domains found in Vav1 that are typical of signal transducing proteins. In fact, Vav1 is a GEF for Rac1, Rac2, and RhoG that becomes active after tyrosine phosphorylation (Tybulewicz et al., 2003[246]). Activated Akt phosphorylates TSC2 at multiple residues (Ser939, Thr1462), disrupting TSC1/2 complex formation and thus TSC2's GAP activity toward the GTPase Rheb (Inoki et al., 2002[106]). Rheb-GTP, liberated from TSC2 inhibition, directly stimulates mTORC1 kinase at the lysosomal surface, where mTORC1 is additionally conditioned on amino acid availability. mTORC1 drives the T cell's anabolic program primarily through phosphorylation of S6 kinase 1 (S6K1, promoting ribosome biogenesis and mRNA translation) and 4E-BP1 (an eIF4E repressor whose phosphorylation releases eIF4E to engage the 5'-cap of mRNAs encoding metabolic enzymes and growth factors) (Reynolds et al., 2004[204]; Huang and Manning, 2009[102]).

mTORC1 functions as a critical metabolic checkpoint that integrates three orthogonal upstream regulatory inputs: growth factor signals (PI3K-Akt-Rheb axis), cellular energy status (AMPK-TSC2 axis), and amino acid availability (Rag GTPase axis) (Dibble and Cantley, 2015[52]; Rehbein et al., 2021[201]; Lama-Sherpa et al., 2023[128]). Under conditions of energy depletion (elevated AMP:ATP ratio), AMPK phosphorylates TSC2 at Ser1387 (potentiating its GAP activity toward Rheb) and directly phosphorylates the mTORC1 component Raptor at Ser792, imposing dual inhibitory pressure on mTORC1 and upregulating autophagy and fatty acid oxidation (FAO) (Agarwal et al., 2015[2]). This tripartite integration ensures that T cells only commit to anabolic effector programs when all three conditions adequate antigen signal, sufficient energy, and amino acid availability are simultaneously met. SLC1A5 (ASCT2)-mediated glutamine import, which is required for Rag GTPase activation and mTORC1 recruitment to the lysosome, is specifically upregulated by TCR stimulation. ASCT2-deficient T cells show restricted clonal expansion and impaired Th1 and Th17 response, demonstrating that amino acid sensing directly regulated the T cell effector differentiation through mTORC1 control (Nakaya et al., 2014[185]).

### 3.5 Signal integration logic

The four downstream signaling axes mentioned above are linked together by various forms of cross-regulatory interactions that make their outputs interdependent as opposed to additive. Reported mechanistically formed cross-talk nodes are: (i) mTORC1-S6K1 feedback: S6K1, which is a downstream regulator of mTORC1, phosphorylates and inhibits TSC1 deregulating mTORC1 (Julien et al., 2010[115]), to generate a negative feedback that naturally limits sustained Akt Ser473 phosphorylation and restricts mTORC2 activity during long-term stimulation a feedback loop that self-regulates and prevents oncogenic Akt hyperactivation (ii) IKKβ-mediated mTOR regulation (Lee et al., 2008[130]): activated IKKβ phosphorylates and inhibits TSC1, diminishing mTORC1 and providing a direct link between the NF-κB pathway and metabolic reprogramming. This also might explain why NF-κB pathway blockade reduces glycolytic metabolism in T cells beyond what PKCθ inhibition alone achieves and lastly (iii) NF-κB-NFAT cross-talk (Guo et al., 2024[89]). Signal strength (determined by TCR-pMHC affinity, antigen dose, and co-stimulatory input), signal duration, and temporal ordering each independently influence T cell fate through mechanisms now well-characterized at the molecular level (van Panhuys, 2016[249]). Signal strength is transduced by the digital/graded hierarchy where strong signals activate both the digital ERK/NFAT switch and generate high-amplitude graded NF-κB and IRF4 responses, while weak signals may activate the digital switch only transiently or fail to achieve sustained NFAT nuclear occupancy, yielding a tolerance or Egr2-driven regulatory gene expression profile (Zikherman and Au-Yeung, 2015[291]). Signal duration is integrated primarily through the Ca^2+-^calcineurin-NFAT axis, which functions as a molecular timer. The Egr2 tolerance gene is sensitive to brief low-affinity stimulation (short NFAT nuclear dwell time), while the effector gene Ifng requires prolonged high-affinity stimulation (sustained NFAT nuclear occupancy)-a temporal encoding mechanism directly measured by intravital imaging. IRF4 integrates both dimensions: it is proportional to signal strength (via NF-κB-driven transcription) and its nuclear accumulation kinetics reflect signal duration (via the delay in Irf4 mRNA induction at weaker stimulations) (Colella et al., 2008[39]). T cells mainly operate as temporal signal processors operating over hours, not merely as instantaneous biochemical detectors positing a conceptual shift with direct implications for CAR-T engineering.

The T cell signaling network simultaneously implements two complementary signal-processing modes: digital threshold detection (preventing activation below a well-defined antigen quality threshold) and analog response encoding (preserving quantitative signal strength information above threshold for fate calibration). These modes are implemented in a hierarchical architecture across pathways. The biological logic of this layered architecture is deeply functional (Zikherman and Au-Yeung, 2015[291]). The digital threshold in NFAT and ERK activation ensures that T cells do not mount productive immune responses to self-antigens or low-level non-specific stimuli maintaining self-tolerance and preventing autoimmunity. Once the digital threshold is crossed and NFAT/ERK are activated, the graded NF-κB amplitude and IRF4 expression encode the quantitative strength of the antigen signal, calibrating the magnitude and qualitative type of the effector response in proportion to pathogen burden and TCR affinity (Wither et al., 2023[263]). High IRF4 drives Blimp-1, T-bet, and SLEC fate; low IRF4 supports Tfh (via Bcl-6 de-repression), memory precursor, or regulatory T cell differentiation. IRF4 therefore functions as the central effector-fate rheostat that translates network-level quantitative signal processing into a lineage-commitment decision, a position at the convergence of the digital threshold-crossing events and the graded response encoders (Duckworth and Groom, 2021[55]).

T cell activation cannot be explained by linear, independent signaling pathways; instead, it operates as an integrated, non-linear network. There are several pieces of evidence that support this. Mainly the transcriptional regulation relies on cooperative logic and key genes such as NFAT, AP-1 and NF-kB must all bind IL-2 to activate this pathway in complete manner. NFAT alone can activate tolerance-related genes (including PD-1), but in combination with AP-1 activates the effector programs, proving that the results are network-dependent and not pathway-dependent. Secondly, the non-linear behavior is brought about by cross-talk and feedback. These interactions generate emergent properties which are not deductible in each case of individual pathways. Thirdly, information is coded in time. ERK and NFAT signaling pathways diverge after a time interval, with the ease of antigen binding and dose being encoded by different variables. Lastly, the cell fate is determined by network balance. NFAT to AP-1 ratio is what determines whether T cells are activated or enter into anergy/exhaustion. When the balance is disrupted in tumors, the effect of exhaustion ensues, whereas treatment interventions such as PD-1 blockade restore the network balance. Lastly, cellular responses are quantitative, combinatorial, rather than binary furthering the signaling patterns. Signal strength, length of signal, and pathway integration determine differentiation into effector, memory, or regulatory states. This requires effective immunotherapies to act on multiple network nodes in parallel, and no longer on the individual pathways.

## 4. Spatiotemporal Dynamics of T Cell Activation

**Figure 2:**
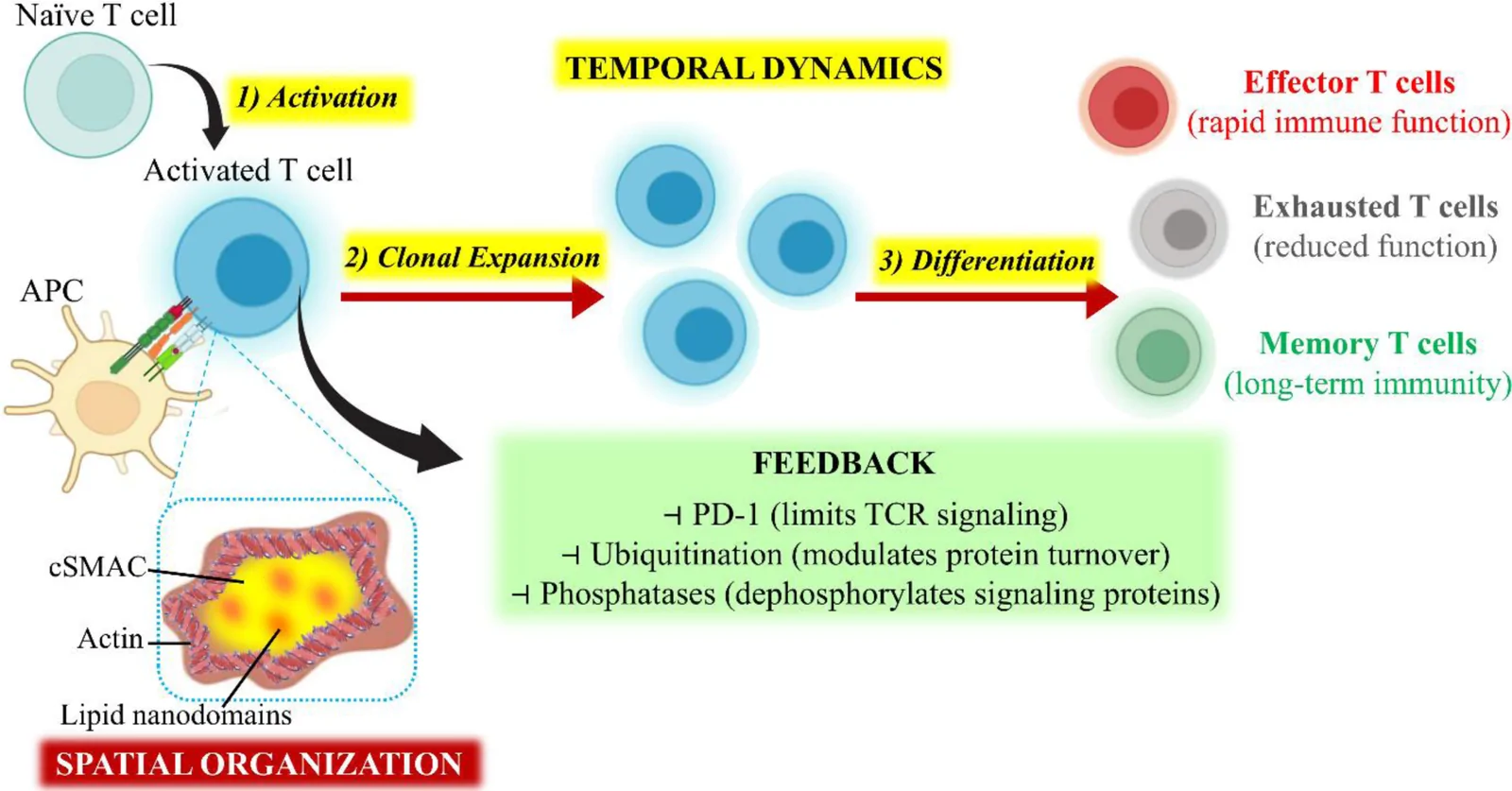
Overview of T cell activation highlighting spatial organization, temporal dynamics, and regulatory feedback. Naïve T cells are activated upon interaction with antigen-presenting cells (APCs), initiating immune synapse formation with structured domains such as the cSMAC, actin cytoskeleton, and lipid nanodomains. Following activation, T cells undergo clonal expansion and differentiate into effector, memory, or exhausted states. These processes are tightly regulated over time and modulated by feedback mechanisms, including PD-1 signaling, ubiquitination, and phosphatase activity, which fine-tune TCR signaling and T cell fate outcomes.

T cell activation is governed not only by the strength of receptor signaling but also by its spatial organization, temporal coordination, and the regulatory feedback mechanisms that collectively shape cell fate decisions (Figure 2(Fig. 2)).

### 4.1 Temporal dynamics

**Figure 3:**
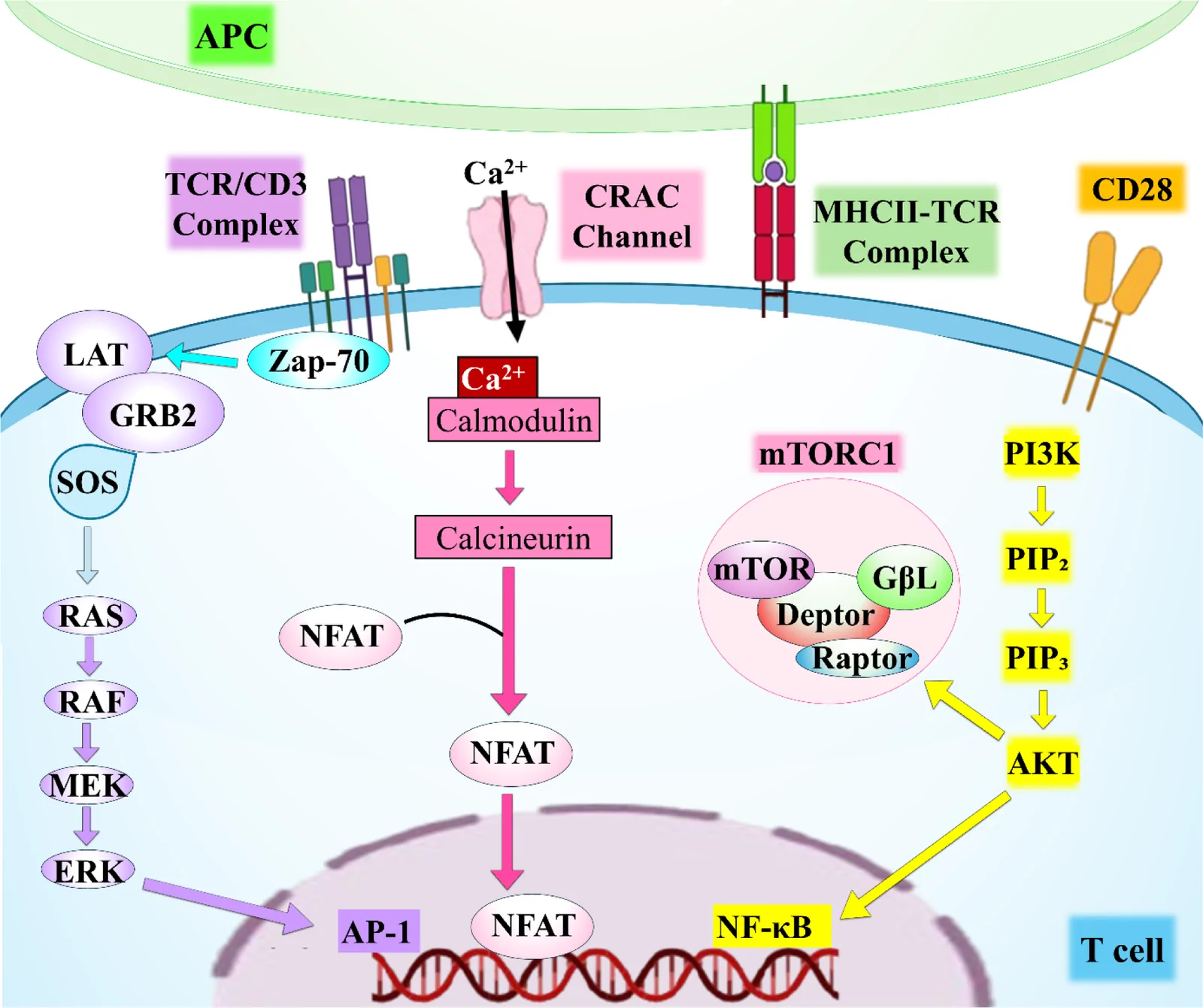
Schematic representation of T cell activation signaling at the immune synapse. Engagement of the TCR/CD3 complex with peptide-MHC II on the antigen-presenting cell (APC) activates ZAP-70 and the LAT signalosome, triggering the RAS-RAF-MEK-ERK pathway and AP-1 transcription. Concurrently, calcium influx through CRAC channels activates the calmodulin-calcineurin pathway, leading to NFAT nuclear translocation. Co-stimulation via CD28 activates the PI3K-PIP3-AKT pathway, promoting mTORC1 signaling and NF-κB activation. These integrated pathways coordinate gene transcription programs essential for T cell activation, proliferation, and differentiation.

T cell activation is regulated by the temporal dynamics that determine the activation of naïve cells into specialized effector, memory, or exhausted cells (Figure 3(Fig. 3)). Early signal stages induce early reprogramming of metabolism and transcription, orients T cells to proliferate, and late signaling stages stabilize lineage commitment and differentiation of functions. These processes are further negotiated, creating stage-specific transient populations of cells through oscillatory patterns of signaling and gene expression contribute to cellular heterogeneity and immune response regulation. Such integrated temporal programs are essential when considering all the aspects of a functional immunity and when trying to maximize treatment responses, such as vaccines, CAR-T therapy, and immune checkpoint blockade. To eliminate these dynamics, integrative proteomic and transcriptomic profiling techniques have been adopted to determine molecular signatures relating to early and late phases of activation. With the help of this strategy, primary human CD4+ and CD8+ T cells activated in vitro using anti-CD3/CD28 beads demonstrated different initial and proliferating stages. The initial activation was characterized by a temporary down-regulation of GLUT1 mRNA and protein, which suggests a rapid metabolic priming, and also by phase-specific uncoupling of transcriptional and translational programs which is reflected by transcriptional divergence although convergent protein profiles (Weerakoon et al., 2024[261]). Based on this knowledge, type I interferons (IFN-I) were reported to coordinate a sequence of transcriptional and epigenetic waves over the course of 96 hours, whereby initial IRF/STAT activity and subsequent AP-1-mediated transcription of coinhibitory receptors including PD-1, TIM-3 and LAG-3 were observed, and TIGIT was suppressed. In this study, the authors emphasized the temporal coordination of signaling programs in influencing functional differentiation and immune checkpoint expression (Sumida et al., 2022[239]). Substantiating these observations, total single-cell transcriptomics of 655,349 CD4+T cells at several time points of cell activation revealed 38 population states in transcriptomes, with transient populations emerging at only one time, highlighting that temporal control of transcriptional state in human immunity produces stage-specific phenotypes and has the power to shape susceptibility to immune-mediated disease (Soskic et al., 2022[234]).

The clonal number and functional performance are also controlled by temporal regulation, in which early or late signals are integrated at a cellular level. In a controlled SARS-CoV-2 challenge, the growth of CD4+ T cells was faster and was followed by contraction whereas clonotypes of the same TCR specificity grow in the same direction, so the kinetics of activation were mainly controlled by the frequency of the progenitor cells but not by the signal strength in the TCR (Pyo et al., 2025[197]). Longitudinal studies have demonstrated the existence of oscillatory transcriptional programmes that control differentiation of CD8+ T cells into effector, memory and exhausted states comprising transient TCF-1/Kitchener-like progenitor-like subsets and Zeb2-regulated exhausted subsets and showing how rhythmic gene expression consolidated functional specialization with time (Giles et al., 2022[79]). These principles were validated by a clinical CAR-T cell study which revealed that the emergence of memory-like CD8+ clones at an early phase was predictive of therapeutic response, and at an late phase, it was the regulation subsets that blocked proliferation, as a consequence of time-regulated and oscillatory signaling dynamics (Haradhvala et al., 2022[94]).

Dynamic aspects on a temporal basis were also captured with respect to tissue specific and disease situations wherein there were functional implications of early versus late signaling. CXCL13+CD8+ T cells and dendritic cell-mediated PD-L1 expression selectively increased in responsive microenvironment in tumors, and temporally regulated inhibitory signals limited early activation of T cells (Liu et al., 2022[153]). The dynamics of T cell activity under temporal modulation were also subject to dendritic cell PD-L1 expression that placed initial inhibitory restrictions conditioning downstream effector differentiation (Peng et al., 2020[194]). Outside of cancer, in Parkinson disease, temporally resolved CD8+ T cell infiltration prior to alpha-synuclein aggregation indicates that an early or late cytotoxic program is established to decide pathological outcome (Galiano-Landeira et al., 2020[69]). In a parallel manner, long-term tumor-wide entrapment of exhaustion programs through tumor-associated macrophage interactions gave rise to spatiotemporal feedback loops which maintained immunosuppression (Kersten et al., 2022[121]). The biphasic responses were further illustrated in the vaccine study, where early activation and memory development was followed by rapid cytotoxic discrimination, which showed the role of signal oscillations to determine protective immunity (González-Fernández et al., 2026[83]). Furthermore, longitudinal studies using triple-negative breast cancer models demonstrated that early cytotoxic and helper T cell visible markers change into IL-17/22-producing subsets at more advanced stages and antigen-presenting cells diminish and immunosuppressive macrophages remain, with stage-related immune fitted and the marked worth of time-regulated interventions (Iftehimul et al., 2026[105]). Together, the studies are convergent and demonstrate that early and late signaling levels and oscillatory dynamic patterns are key predictors of T cell fate, functional heterogeneity, and therapeutic potential offering a conceptual paradigm of immunotherapy and vaccine design.

### 4.2 Spatial compartmentalization

Spatial compartmentalization is one of the core concepts of T cell activation and it is necessary to be sure that the signaling events are triggered, spread, and shut down in a highly mechanized fashion. At plasma membrane, the engagement of TCR starts with the formation of peripheral microclusters that maintain early signaling and sequentially rearrange into central supramolecular activation clusters that act as receptor sorting hubs and signal attenuation parts (Varma et al., 2006[250]). This spatial development maintains the unrestricted diffusion of the signals and offers an opportunity to control T cell responses by precise modulation. These clusters are organized by preexisting membrane nanodomains that serve as a structural scaffold, and upon stimulation binds signaling molecules rapidly and assembles them into a cluster (Dinic et al., 2015[53]). Additional experimental manipulations of the geometry of the membrane also indicated that nanoscale changes in both the volvometry and topology of the membrane can regulate the localization and interaction of TCR-associated proteins, underscoring the role played by physical properties of the membrane in determining signaling fidelity (Kai, 2015[117]).

These signaling domains are interconnected to be sustained and enhance functional efficiency by the coordinated action of the cytoskeleton and lipid-mediated mechanisms. Actin filaments keep TCR microclusters intact, which leave higher-order organization and the vital protein-protein contacts needed to support efficient signal transmission intact (Vámosi et al., 2024[248]). The lipid composition and especially PI(4,5)P2 have been shown to control the process of recruiting actin remodeling proteins and the spatial distribution of signaling complexes and disruption in lipid organization has been demonstrated to disrupt the process of cytoskeletal dynamics and to inhibit the process of T cell activation (Hou, 2014[99]). Spatial positioning of co-receptors in these areas also enhances signaling by amplification, e.g., having SLAMF6 and the CD3 complex co-localize enhances downstream TCR signaling, which shows that nanoscale scale receptors structure can tune their functional responsiveness (Gartshteyn et al., 2023[74]).

This process of spatial compartmentalization is not just limited to the plasma membrane, but also intracellular and tissue-level organization, can be described as the connection between molecular localization and functional outcomes. CD59-regulated intracellular trafficking of Ras causes components of the MAPK pathway to be targeted to particular subcellular sites to regulate T cell proliferation and effector functions (Li et al., 2022[143]). At the tissue level, the T cell-produced cytokines, like IFN-γ, generate localized gradients that selectively counteract immunosuppressive pathways and distinguish between local and global signaling actions and coordinate immune responses on complex tissue landscapes (Hoekstra et al., 2024[97]). T cell responses in tumors and stromal areas that are compartment-specific further define antitumor response and also determine therapeutic performance (Sadeghirad et al., 2023[211]). These observations highlight that spatial compartmentalization is a multiscale regulatory circuit, whose proper operation assimilates membrane organization, intracellular signaling as well as tissue architecture to regulate the quality and magnitude of T cell responses. In general, spatial compartmentalization offers a hierarchical dynamic structure through which T cells attain functional accuracy and flexibility. This framework by organizing nanodomains to tissue-scale niches provides essential insights into immunoregulatory mechanisms and provides key insights into how to make T cells respond to various stimuli, which is essential in immunotherapeutic intervention strategies.

### 4.3 Signal termination and feedback

Termination of a signal and negative feedback are necessary to provide specific T cell response without excessive immunity or hyperactivation and autoimmunity. This process is supported by a complex web of interactions between kinases and phosphatases, ubiquitin-mediated pathways, and a pathway of inhibitory receptor turnover all of which lead to the spatial, temporal and functional regulation of TCR signaling. It was shown that Zap70 plays a key role in the activation of naïve, effector and memory T cells, whereas the activation of integrin signaling by Rap1, and T cell suppressive activity was also activated without the catalytic activity of Zap70, which highlights branch-specific, modular regulation in TCR signaling pathways (Au-Yeung et al., 2010[9]). Ubiquitin-dependent feedback also limits effector responses: UBA6 deletion in T cells was observed to stimulate IFN-γ production by CD4+ and CD8+ T cells, and result in multi-organ inflammation proving the ubiquitination is a negative checkpoint to prevent pathological immune activation (Lee et al., 2022[134]). Ubiquitin-dependent turnover is also tightly regulated by inhibitory receptors, with CTLA-4 degraded by using K27-, K29-, and K63-linked polyubiquitin chains, and deubiquitination of the inhibitory receptor controlled by USP8, which also indicates a reversible process where inhibitory signaling is dynamically regulated (Tey et al., 2024[242]).

A further control, fine-tuning signal strength, adhesion and functional outcome is achieved through spatial and temporal compartmentalization at the immunological synapse. Phosphatases and ubiquitin ligases play their roles in specific microdomains to tune TCR-proximal signaling. DUSP22 dephosphorylated Lck to suppress TCR activation, but UBR2 increased Lys63-linked ubiquitination of Lck to create a negative feedback loop, to maintain signal propagation equilibrium (Shih et al., 2024[225]). One of the studies has observed that C3G was recruited to the peripheral SMAC by CrkII adaptor proteins, which increased Rap1 activation and LFA-1-dependent adhesion, whereas SHP-1 dephosphorylated CrkII at the central SMAC, which regulated its localization and activity (Azoulay-Alfaguter et al., 2017[10]). Moreover, CrkII also acted as a scaffold to Cbl which ubiquitinated and degraded C3G following TCR/CD3 interactions, thereby giving a temporally precise negative feedback that restricted how much signal was generated and the duration (Nath et al., 2023[186]).

Molecular feedback between non-degradative ubiquitination and orchestration of spatial receptor patterns regulate T cell functional and therapeutic responses. ARIH1 facilitated the infiltration of cytotoxic T cells and reinforced response to PD-L1 blockade by ubiquitin-mediated degradation of DNA-PKcs, triggering STING signaling (Liu et al., 2023[155]). Using this regulatory principle, the highlight of proteomic analyses was the wide scale non-degradative ubiquitination of primary CD4++ T cells in the form of K29-, K33-, and K63-linked ubiquitin chains that were necessary to modulate TCR signaling without the need of proteasomal degradation (Dybas et al., 2019[60]). Negative feedback also occurred using USP24, which kept PD-1 stable in CD8+ T cells and restrained effector activity cells in chronic stimulator settings (Hsieh et al., 2025[100]). Conversely, KLHL6 maintained transcriptional and mitochondrial responses in chronic stimulated T cells which maintained effector activity and overcame exhaustion (Chen et al., 2026[34]). Spatial organization also had an additional effect, amplifying T cell responses: anti-FcRH5/CD3 bispecific antibodies catalyzed TCR signaling and cytotoxicity through clustering target molecules and avoiding inhibitory phosphatases (Li et al., 2017[142]). Taken together, these studies show that the T cell signal termination is regulated by a complex network of ubiquitin-mediated feedback, phosphatase activity, and space receptor compartmentalization, and the connection between the mechanistic control and immune response and therapeutic prospective.

## 5. Experimental Platforms to Study T Cell Activation

### 5.1 Reductionist vs. physiological systems

Cellular models of T cell activation can be as simple as the reductionist models of antibody-coated beads, soluble complexes, or supported lipid bilayers, which provide fine control of TCR phenotype engagement and co-stimulatory inputs, or tissue engineered models of APCs and other physiologically-realistic systems, which incorporate both antigen presentation, co-stimulation, and cytokine ligands. Such systems make it possible to study T cell proliferation, differentiation, effecter function, and synapse formation as well as mechanotransduction in a controlled or semi-physiological environment. As seen in the studies summarized in Table 1(Tab. 1) (References in Table 1: Choi et al., 2021[37]; Dam et al., 2021[44]; Dudaniec et al., 2021[58]; Hamieh et al., 2021[92]; Jassin et al., 2026[110]; Kongkaew et al., 2022[122]; Lacouture et al., 2024[125]; Leithner et al., 2026[136]; Li et al., 2019[141]; Litvinova et al., 2021[150]; Lustig et al., 2020[163]; Meås et al., 2025[172]; Reithofer et al., 2021[202]; Ren et al., 2019[203]; Saliba et al., 2019[212]; Sharkeev et al., 2021[220]; Singh et al., 2022[231]; Tvingsholm et al., 2023[245]; Vidard, 2023[251]; Yang et al., 2025[273]; Zhao et al., 2019[287]), this variety can be seen not only in terms of the classification of the system type of each of these platforms and their physiological relevance and uses, but also in their more general subdivision into categories based on basic immunological research and the development of T cell-based immunotherapy. Table 1(Tab. 1) is intended as a practical decision-support framework that assists investigators in selecting experimental models according to the biological question, desired physiological relevance, scalability, and translational applicability.

### 5.2 Advanced imaging technologies

Improved imaging systems and experimental characterizations have changed how T cells are studied because they allow the visualization of these immune cells in real time, starting with subcellular signaling events up to system-wide behaviors. These receptors show how the T cells combine the environmental signals in order to make the activation decision, to differentiate as well as to perform effector functions. As an example, BEHAV3D allowed real-time 3D imaging of T cells in monoculture with patient-derived organoids, which enables observing dynamic behavior, including cell migration, synapses, and cytotoxicity, leading to the discovery of functional heterogeneity at the level of single-cell analysis (Alieva et al., 2024[5]). On the receptor level, four dimensional lattice light-sheet microscopy enabled a detailed tracing of TCR microclusters on the surface of live primary T cells revealing how spatial rearrangements store information on activation strength and signaling outcome (Rosenberg et al., 2020[208]). The next step to translate these observations to the entire organism was that PET imaging using a T cell-specific radiotracer assessed activated T cells in the various organs after SARS-CoV-2 infection showing that T cell activation is systemic and local cellular events correlate with long-term immune outcomes (Peluso et al., 2023[193]).

Using experimental imaging platforms at the tissue level, we have discovered the nature with which local microenvironment and dynamic cell-cell interactions shape T cell activation. The intravital calcium imaging showed that autoreactive CD4+ T cells in the intestinal lamina propria underwent microbiota-dependent calcium signals, which triggered differentiation into Th17-like phenotypes followed by migration to the inflammatory sites (Bauer et al., 2023[14]). Experimental autoimmune encephalomyelitis using two-photon microscopy revealed seasoned Th17 cells that secreted IFN-γ switched to Th 1 types locomotion in inflamed tissue, showing a functional plasticity in T cells following activation (Loos et al., 2020[159]). Moreover, motility of naive CD4+ T cells in relation to various depths of the tissues during inflammation was tracked by three-photon images of lymph nodes, and the spatial microenvironmental cues influenced activation, migration and positioning in lymphoid architecture (Choe et al., 2022[36]). Modern light-sheet imaging was able to apply these findings to more disease-relevant models by tracing T cells within tumor microenvironment in the context of immunotherapy and shown how the dynamics in localized activation results in coordinated population behavior (Wang et al., 2021[255]).

At the subcellular and molecular levels, high-tech imaging methods have torn down the structural processes involved in the stimulation of T cells. Using imaging to visualize the process of immunological synapse formation has shown that the vesicular trafficking process and polarized delivery of cytokines and lytic granules are orchestrated by actin and microtubule cytoskeleton remodeling required in T cell effector activities (Juzans et al., 2023[116]). Super-resolution microscopy also showed that nanoscale organization of CAR molecules on engineered T cells had a direct impact on the potency of activation and cytotoxic ability (Gehrke et al., 2025[77]). The imaging of single-molecule CD4 receptors revealed that ligand-induced clustering is an initial stimulator of signaling, which connects receptor nanoscale activity to subsequent downstream phenomena (Yuan et al., 2021[281]). It was confirmed by high-resolution tracking TCR microclusters in 3D that the receptor mobility and spatial composition encode the magnitude of activation as well as functional outcome (Rosenberg et al., 2020[208]). In combination, these works emphasize the means by which sophisticated imaging modalities can mechanistically inform T cell activation between receptor arrangement and biologic delivery, and as such, experimental interventions to T cell behavior can be asked, which can be enabled and subsequently inform immunotherapy design or precision-targeted interventions.

### 5.3 Quantitative signaling approaches

Mechanistic analyses of T cell activation are progressively based on quantitative platforms which couple receptor level mechanics to intra-cellular signaling and eventually to functional responses. The physical forces acting on the TCR during antigen recognition have been attributed to active hypotheses to tune activation thresholds, but untested before. TCR-imposed tensile forces were quantitatively measured using FRET-based molecular force sensors immobilized to planar supported lipid bilayers using them, and a characteristic peak tensile force of ~5 pN was measured on gel-phase SLBs with lower tensile forces observed on fluid-phase SLBs (Göhring et al., 2021[81]). This data showed that mechanical tension is not as much of a by-product of ligand binding but is as such, an active organizer of signaling thresholds, inspiring more studies to take the next step to intentionally manipulate these forces. This was overcome by optogenetic platforms based on light-sensitive phytochrome B receptor, in which the length of time TCR-ligand binding is controlled with precision in milliseconds, demonstrating that downstream kinase activation is controlled not by a fixed affinity but by ligand half-life, empirically proving the kinetic proofreading model (Yousefi et al., 2019[279]). Furthermore, immunological synapse (IS)-based FRET-based biosensors of mesothelin in mesothelin-targeting CAR-T cells demonstrated that synapses are primarily controlled by robustness, as opposed to optimal ligand-binding capacity, and that mesothelin-targeting receptor-based mechanics are directly linked to T cell functional capacity (Lee et al., 2025[131]).

These receptor-level cues are then programmed into functional programs through intracellular signaling cascades in T cells. High-resolution biosensors of FRET in T cells demonstrated that interaction of stimulant receptors rapidly triggers ZAP70 and ERK, and co-stimulant environment regulates the degree and dynamics of these messages (Zhou et al., 2022[289]). A reconstitution system based on IFNAR1/2 allowed quantitative measurement of cytokine released: secretion of IL-2 and IFN-γ was found to directly depend on subtle differences in receptor signaling in activated T cells (Hayashi et al., 2024[96]). The co-immunoprecipitation experiment also characterized the assembly of T cell signalosomes, with both conserved TCR modules and specific interactions that determine specific functional responses (Ritmeester-Loy et al., 2024[206]).

The T cell responses should be viewed in physiologically significant contexts of interaction of chemical, mechanical and spatial stimuli. Using microfluidic platforms, in real time during flow and chemokine gradients, T cell adhesion, chemotaxis and migration across endothelium were examined; it was revealed that migratory behavior was directly dependent on activation state (de Haan et al., 2021[48]). The tumor-on-a-chip systems also revealed that the infiltration of T cells is increased in hypoxic areas and regulated by the interaction of monocytes and cancer cells indicating that the recruitment of immune cells is fine-tuned by microenvironmental cues (Aung et al., 2020[8]). Mechanical confinement in microchannels revealed that the velocity of T cells and migration of CAR-T cells during mechanical tumor-like confinement varied with intracellular activation and mechanical obstacle (Zhang-Zhou et al., 2025[286]). These observations were combined into high-dimensional single-cell profiling that showed that T cells differentiate to resemble naïve or effector memory cell-states, with effectorness gradients that are associated with cytokine responsiveness and migratory ability (Cano-Gamez et al., 2020[24]). Collectively, the techniques span three orders of magnitude connecting molecules to cells and tissues to provide a detailed, quantitative approach to the drugs of how the T cell activation, signaling, and functional behavior are integrated to respond to complex environments.

### 5.4 High-dimensional immune profiling

High-dimensional immune profiling has become a revolutionary experimental system used to analyze T cell activation, allowing the specific characterization of heterogeneity in phenotype, functional and metabolic measures of health and disease. To map the T cell differentiation pathways, as well as functional states, these methods combine multi-parameter cytometry, single-cell RNA and protein profiling, and computational analysis. As an example, analyzing the CD19 CAR-T cells with a 16-plex microfluidics-based cytokine platform identified clear polyfunctional subsets of anti-tumor, stimulatory, regulatory, and inflammatory functions, with computational tools enabling the donor-specific visualization and prediction of therapeutic potency and safety (Xue et al., 2017[270]). Using equivalent platforms in autoimmune systems, mass cytometry of peripheral immune cells single cell engravers of severe aplastic anemia have demonstrated the importance of the γδT17 cells as drivers of autoreactive T cell activation, and the production of IL-17A associated with the severity of the disease, making biomarker discovery through single-cell mass cytometry highly valuable (Wang et al., 2023[256]). The mass integration of single-cell RNA sequencing, T/B cell receptor sequencing and mass cytometry in 220 healthy participants showed that age- and tissue-specific rewiring among T subset cells, GNLY+ CD8+ effector memory and MAIT cells, is observed under high-dimensional platforms that offer a general framework to determine immune competencies by various studied ages (Wang et al., 2025[259]).

In addition to giving a picture of the normal and pathogenic T cell states, these platforms have elucidated how therapeutic interventions alter T cell function. In Crohn disease, CD8+ IL-17-producing Tc17 cells were increased in blood and in the intestinal tissue, exhibited a unique CD6high, CD39, CD69, PD-1 and CD27low signature, and in vitro target CD6, reduced IL-17, IFN-γ and TNF production, which is directly linked to the pathology of the disease (Globig et al., 2022[80]). On the same note, T cell composition during therapy with belimumab in systemic lupus erythematosus changed by increasing regulatory T-like and naïve cells and changing Th17/Treg ratios, also showing how profiling can identify subtle changes instigated by therapy (Maeda et al., 2023[167]). A 36-player spectral flow cytometry panel in CAR-T production has shown that early mid-expansion superfood solutions held stem-like CD4+ Th1 cells, but high-dimensional monitoring revealed that extended culture increased the proportion of terminally differentiated CD8+ Tc1/NK-like cells, indicating that high-dimensional monitoring affects ex vivo optimizing T cell functional capacity (Cadinanos-Garai et al., 2025[23]).

Mechanistic understanding of the role of metabolic, epigenetic, and tissue-specific factors in the activation of T cells has also been gained using high-dimensional platforms. The dynamic interaction of metabolism and effector function was involved in the dynamics of mass cytometry of CD8+ T cells during Listeria monocytogenes infection, with glycolytic and oxidative phosphorylation programs reaching their peak during the fifth day of infection and maintained thereafter, suggesting this pattern to be conserved in CAR-T cells (Levine et al., 2021[138]). Combination of scATAC-seq and scRNA-seq in severe COVID-19 showed disrupted Th1 CD4+ T responses and hyperstimulated CD8+ T cells, and associated epigenetic restructure to dysregulated immune conditions (Li et al., 2021[144]). The CITE-seq analysis further added a powerful resolution when it combined the protein and transcriptomic data to reveal previously unknown functional diversity (Hwang et al., 2024[103]). Compartmentalized states of activation in single cells through tissue specific single cell RNA sequencing of lungs, lymph nodes, bone marrow, and blood served as a reference framework to understand the tumor-infiltrating lymphocyte states (Szabo et al., 2019[240]). Based on these findings, in patients with lung adenocarcinoma, single-cell RNA sequencing of tumor-infiltrating lymphocytes cocultured with autologous tumor cells in patients has identified that T cells show heterogeneous activation and effector phenotypes, in which selection based on single markers is not sufficient to isolate tumor-reactive TCRs. TCRs capable of specifically recognizing autologous tumor cells were successfully identified in hepatocellular carcinoma by calculating a composite activation score of several cytokines and effector molecules such as IFNG, IL2, TNF, IL2RA, CD69, TNFRSF9, GZMB, GZMA, GZMK, and PRF1 (Zhang et al., 2024[283]). While in hepatocellular carcinoma, LDHA-mediated lactate promoted Th1 differentiation and enhanced JAML+ CD8+ cytotoxicity, and agonistic anti-JAML therapy reduced tumor burden and prolonged survival (Chen et al., 2025[33]). This research showed how high-dimensional experimental systems mediate mechanistic conceptualization of T cell persist in linking mechanistic understanding of T cell activation to translational application and discuss why such experimental systems form the core of current immunological investigation and therapeutic development.

### 5.5 Integrative methodologies

Under the experimental platforms to analyze T cell activation, integrative methodologies offered a holism perspective in respect of a combination of signaling, metabolism and effector functions as a whole culminating into the T cell responses. Such methods allowed mechanisms of temporal and mechanistic analysis of the process of activation, which presented the interaction between early signaling cascades and downstream metabolic reprogramming. As an example, proteomics and phosphoproteomics with multiplexed isobaric labeling made it possible to map the protein expression and phosphorylation dynamics of TCR stimulation, showing that early and rapid phosphorylation events were followed by slow ribosomal biogenesis and mitochondrial activation and that oxidative phosphorylation controlled by COX10 was a central driver of T cell activation (Tan et al., 2017[241]). It is developed further with the observation that ex vivo stimulation of human CD4+ and CD8+ T cells using anti-CD3/CD28 beads and temporal proteomic and transcriptomic profiling showed that early GLUT1 downregulation is regulated by both subsets, but convergent patterns of protein rewiring, demonstrating that metabolic and molecular rewiring are central to proliferation and early effector differentiation (Weerakoon et al., 2024[261]).

On top of molecular profiling, high-dimensional single-cell platforms have identified the heterogeneity and plasticity of T cell functional states. The use of mass cytometry (CyTOF) with activation-induced marker assays, intracellular staining of cytokines, and multiplex barcoding made a simultaneous evaluation of the antigen-specific T cell responses possible, disclosing that optimized dual-marker combinations could be trusted to trace the cytokine-producing populations (Wan et al., 2026[254]). Applying these conclusions to the pathological scenarios, mass cytometry, single-cell RNA sequencing, TCR sequencing and tissue imaging were used to investigate chronic lymphocytic leukemia, revealing a plethora of exhausted T cell phenotypes, such as precursor and terminally exhausted cells, and identifying galectin-9 as a potential therapeutic target (Llaó-Cid et al., 2025[157]). These findings inspired analogous combining techniques in CAR-T cell studies in conjunction of 3D lmCT/bioluminescence tomography, light-sheet fluorescence microscopy, and cyclic immunofluorescence, which are generally used to monitor CAR-T cell transit, tumor penetration, and active modulation. This demonstrated perivascular and tumor-periphery localization of preferential accumulation and the association of spatial localization with functional consequences (Pfeifer et al., 2022[195]). The monitoring of 4-1BB-mediated CD19 CAR-T cells by using single-cell RNA and protein profiling further showed movement into nonproliferative and highly differentiated and exhausted states and that TIGIT blockage has the potential to stimulate their functional performance (Jackson et al., 2022[108]). The profiling of single cells also revealed hybrid TH1/TH2 cytokine programs and regulation-like behavior without classical differentiation, where integrative platforms have the power to obtain functional plasticity and heterogeneity in therapeutic scenarios of interest (Xhangolli et al., 2019[267]).

These findings were applied to immune control in infection, autoimmunity and systemic inflammation by integrating multi-omics and computational methods. Single-cell transcriptome and antibody sequencing of COVID-19 patients found an abnormal T cell activation, rapid switching between subtypes, and sustained post-recovery inflammation responses, whereas computational modeling revealed predictive markers of disease severity (Chattopadhyay et al., 2022[31]). Longitudinal analyses also revealed early activation of bystanders without systemic inflammation by CD8+ T cells to be related to mild disease whereas delayed activation under inflammatory conditions led to severe effects, suggesting the significance of T cell response temporal dynamics (Bergamaschi et al., 2021[19]). Furthermore, Hi-C chromatin orientation profiling, ATAC-seq, and RNA sequencing in autoimmune disease associated GWAS variants with regulatory elements and transcriptional product, determining causal genes and maladjusted pathways (Shi et al., 2025[223]). In a similar manner, a single-cell and multi-omic study in sepsis discovered seven fundamental regulating genes, and broken signaling networks that propagated immune depletion and a malfunction in T cell interaction (Li et al., 2025[145]). A multiscale quantitative systems pharmacology (QSP) framework was also created that simulates CAR-T cell dynamics in solid tumor, combining antigen recognition, activation and proliferation, phenotype changes, biodistribution and tumor cytotoxicity. The model was calibrated using preclinical and clinical data and simulated virtual patient populations and forecasted therapeutic results in differing dosing regimens. It also occurred the impact of heterogeneity of tumor microenvironment, antigen density, and cytokine milieu on CAR-T persistence and efficacy, which were mechanistically aligned to optimize the trial design, customize therapy, and predict possible resistance in advance (Yang et al., 2025[274]).

All those advances indicate that future studies on T cell activation should embrace a more integrated experimental ecosystem that involves single-cell multi-omics, spatial profiling, longitudinal imaging, and mechanistic computational modeling (Wu et al., 2024[265]). These types of networks facilitate links between receptor-proximal signaling events and transcriptional, metabolic, and functional consequences, spanning multiple biological levels. Furthermore, integrated approaches offer a way in which the different technologies independently deployed can cross validate each other, thus enhancing the reproducibility and confidence in any observation that is biologically meaningful. From a translation point of view, integrating multi-omics data with quantitative systems pharmacology and digital-twin paradigms could eventually facilitate the prediction of individual immune responses and the best-tailored immune therapies.

## 6. Functional Outcomes from Signaling to T Cell Fate

### 6.1 Transcriptional programming

Signal recognition by T cells is converted into transcriptional programmes that determine the functional fate of T cells based on their TCR, co-stimulatory molecules, and the surrounding microenvironment. It is these programs that dictate whether T cells will become effectors, long-term and memory cells or undergo states of functional exhaustion and, thus, influence immune competence and therapeutic potential. The knowledge of how the signal transduction pathways are translated into gene expression patterns is essential to define the mechanism that regulates T cell responses and provides the possibility to therapeutically alter this regulation. The arrangement and strength of signals have a deep impact on determining transcriptional levels in T cells. In CAR-engineered T cells, the pathway signaling CD3ζ signal resulted in a core activation mark, whereas 4-1BB co-stimulation evoked an independent transcriptional program relating to heightened HLA class II and IL-21 genes and diminished PD-1, which favored active over tired activation (Boroughs et al., 2020[20]). Likewise, colonized TCR signal strength and ITK- kinase activity intervened with the NF-κB and AP-1-generated transcription of CD8+ cells with links connecting the proximal inducing precursor to chromatin accessibility and expressing effects on effector genes (Gallagher et al., 2020[71]).

Memory formation and regulatory restraint are also incorporated in the transcriptional networks. AP-1 and NF-κB has been shown to prime the memory CD4+ T cells to recall quickly, showing that transcriptional access and epigenetic modification produce quick, situation-adapted responses (Shittu et al., 2026[228]). Another mechanism, with negative controls like SENP1-NR4A1, is used to regulate a premature effector differentiation checkpoint plotted between functional activation and homeostatic control of T cells (Jiang et al., 2026[112]). Specific modulators such as PARP-1 to context-dependent processes also exemplify the preferential regulation of transcriptional outputs to demonstrate how T cells can adapt to certain environmental and pathogen-associated environmental factors (Gutierrez and Llano, 2025[91]).

The dynamic heterogeneity of transcriptional programs in chronic or specialized environments has been found using high-resolution and multi-omic analysis. The neoantigen-specific CD8+ T cells in tumor cured suboptimal activation and increased inhibitory checkpoints as they showed partial exhaustion response to persistent antigens exposure (Caushi et al., 2021[27]). This observation led to exploration of progenitor-exhausted CD8+ T cells, where TCF-1 has been demonstrated to control early fate decisions, inhibiting terminal KLRG1Hi differentiation in favor of KLRG1Lo precursors via T-bet-to-Eomes transitions and c-Myb-regulated Bcl-2 expression (Chen et al., 2019[35]). Such measures also revealed maternal Preg-TEX CD8+ T cells resting with exhaustion-like characteristics mediated by NFAT-dependent and independent, which served as examples of physiological tolerance adaptation of transcriptional circuits (Lewis et al., 2021[139]). Multi-omic analysis through Vivo-seq also revealed that ERK1/2 and c-FOS phosphorylation marked Th17 cells to produce lineage specific cytokines and bridged early signaling events and functional specialization (Fortmann et al., 2025[66]). Moreover, a set of integrated transcriptional and epigenetics atlases charted differentiation programs in naive vs. memory, effector vs. exhausted T cells and found regulatory factors that can control the expression of effector genes, and facilitate targeted cellular engineering (Giles et al., 2022[78]).

### 6.2 Metabolic reprogramming

T cell metabolism is a principal controller of functional performance transforming environmental signals and intracellular conditions into protections buying decisions on multiplying, differentiations and effecter activity. Instead of just relating energy, the metabolic pathways play an active role in forming the lineage commitment and functional specialization. Glycolysis, oxidative phosphorylation (OXPHOS) and lipid metabolism are dynamically linked to transmit rapid adjustment of energy needs, tissue-specific biosynthetic requirements to activation, proliferation or tissue needs. These circuit cards constitute a significant interface between signaling events and T cell fate offering both functionality and therapeutics.

The initial activation of T cells causes intrinsic reprogramming of metabolism to match energy generation with the requirement of biosynthesis. The engagement between TCR and pyruvate in CD4+ T cells via PDHK1 triggers the increased translation of cytokine mRNAs to the advantage of cytotoxicity and without any effect on the process, showing that an early signal directly rewards lactic adaptations (Menk et al., 2018[175]). This shift in glycolysis complements the energy producing thermogenic mitochondrial OXPHOS that cites Th17 differentiation, and blockage of the ATP synthase diverts cells into Foxp3+ Tregs, evidenced by the fact that the specification of immune lineage identity is governed by energy flux activities as a form of molecular cue (Shin et al., 2020[227]). The mTORC1-PPAR7 axis governs fatty acid metabolism, which provides vital substrates to support the proliferation of naïve and memory T cells combining nutrient availability with signaling networks to enable complete functional differentiation (Angela et al., 2016[6]). Lipid-derived metabolites also participate in immune regulation. Recent studies demonstrate that ceramide signaling through the FPR2 receptor modulates metabolic pathways and cellular energy expenditure, emphasizing the growing importance of lipid signaling in immune-cell metabolism (Lin et al., 2025[148]).

The extrinsic signals also elaborate intrinsic metabolic conditions to differentiate and maintain. Predictably, STING signaling in CD8+ T cells preserved mitochondrial health and mitochondrial decrease, leading to metabolic flux and functional outcomes, and the loss thereof resulted in excess glycolysis, mitochondrial depletion, and malfunctioning effector functions (Xu et al., 2025[269]). STING proves controversial in CD4+ T cells control TH1 and TH9 development via IRF3 /type I interferon - and mTOR-mediated proxies, which demonstrates how extrinsic immune inputs and converge with metacometabolomics styles to drive a subset-specific response (Benoit-Lizon et al., 2022[18]). The metabolic modulation in response to cytokines was observed in expansion of Tregs using IL-7 and IL-15 that favored the glycolytic metabolism and memory stem-like phenotype, improving their survival and functional regulation in xenogeneic graft-versus-host disease in vivo (Filoni et al., 2025[64]).

The effect of therapeutics and long-term T cell activity are also determined by metabolic programming. In CAR-T cells, mitochondrial biogenesis and AP1-dependent transcription were inhibited by tumor-linked stressors, including CD58, to hamper proliferation and cytotoxicity, and inhibition of DUSP6 specifically induced metabolic fitness and revitalization of effector machinery (Ma et al., 2026[164]). The interaction between transcriptional control and metabolic adjustment was shown by transcription-metabolism axes such as LXR2 and non-canonical NF-kB signaling that established stem-like TCF1+ populations and amplified the cytokine-producing ability (Lim et al., 2026[147]). The delivery of RN7SL1 induced effector-memory differentiation, suppressed suppressive myeloid populations, and increased tumor-specific T cells and revealed approaches to overcoming the metabolic constraints of adoptive cell therapy (Johnson et al., 2021[114]). More so, SARDH metabolic checkpoint mediated one-carbon metabolism and epigenetic programs that enhanced cytotoxicity and presented prospects to optimize immune responses to cancer, autoimmune, and inflammatory environments (Si et al., 2025[229]).

### 6.3 Differentiation pathways

The differentiation of T cells is a dynamic and complicated process that involves a combination of inherent transcriptional programs and external environmental and cytokine inputs to produce effector, memory, regulatory or exhausted subsets. Mixture of naive T cells forms premature biases of particular differentiation pathways, which are cultivated by transcription factors, signaling systems and tissue-generated hints to affirm long-term functional possibility. To this end, computational reconstructions of in vivo differentiation patterns showed that naïve CD4+ T cells took different routes to effector or precursor central memory statuses with a subpopulation uniquely sensitive to IFN-I during viral infections and IFN-I-mediated autoimmune responses, demonstrating that early environmental signals play a critical role in the lineage commitment (Deep et al., 2024[49]). FOXO1 is an essential controller of T cell fate that induces memory differentiation but tapers exhaustion-related programs. FOXO1 disruption influences T cells T to adopt exhausted phenotypes, yet maintain its activity in its memory-like phenotype and functional survival under prolonged stimulation conditions (Doan et al., 2024[54]). Likewise, progenitor-like CD8+ T cells separated early in relation to memory progenitors, increasing the expression of TOX-dependent transcriptional models and active histone silences, which permitted self-renewal and sustained reactivity throughout extended antigen presentation, creating an intermediate condition between activation and exhaustion (Yao et al., 2019[275]).

Sustained antigen presentation also facilitated CD8+ T cell differentiation with stable and hierarchical patterns, giving rise to intermediate and terminally exhausted subpopulations with different functional abilities. The intermediate exhausted cells briefly re-acquired the effector capabilities and remained responsive to the PD-L1 blockade, indicating the existence of a continuum between memory and terminal exhaustion (Beltra et al., 2020[17]). The neoantigen- targeting CD8+ T cells in tumor biology had stem-like memory, progenitor exhausted, and terminally exhausted populations; the progenitor exhausted cells retained clonal diversity and produced downstream effector and exhausted ones whereas tissue localization governed progression to terminally exhausted populations (Luo et al., 2026[161]).

Tissue derived signals and cytokines also altered CD4+ T cell plasticity. During cholestatic liver disease, the accumulation of bile acids reprogrammed Tregs to a Th17-like-like phenotype, which inhibits their anti-inflammatory and anti-fibrotic actions (Kudira et al., 2025[124]). Neonatal Tregs promoted tendon repair by the inhibition of IL-33-induced dysregulated inflammation, recruitment of tenocytes, and structural regeneration, indicating that developmental status and tissue conditions can stimulate Treg differentiation to respond in beneficial ways (Arvind et al., 2025[7]). Fate choices mediated by cytokines also affected helper T cell subprograms: TGF-B stimulated TFH differentiation in vitro via c-Maf, that formed a molecular switch between TFH and Th17 fates (Chang et al., 2024[29]), and integrated signals via TCR, TGF-BR and IL-6R stimulated Th17 versus Treg differentiation using alternative SMAD3 complexes, stabilizing the inflammatory or suppressive programmes (Prado et al., 2021[196]). In addition, the sequence of waves of TCR signaling during early thymid T cell development confirmed the temporal specification of CD4+ versus CD8+ lineage via a TCR -calcium-NFAT-GATA3 axis modeling how time-dependent activation female built long-term lineage specifications (Steier et al., 2023[237]). These reports explain how intrinsic transcriptional programs, hierarchical developmental signals and extrinsic signals come together to determine T cell differentiation to ensure that they can be functionally versatile in a variety of physiological and pathological environments.

### 6.4 Heterogeneity and plasticity

Determination of T cell fate is not a linear or uniform pathway but, rather, controlled by extensive heterogeneity and plasticity that do arise during and following activation. There are varying transcriptional, epigenetic and environmental signalings and T cell identity can be constantly remodelled and thereby dynamic transitions between functional states takes place. The heterogeneity especially occurs in memory and effector compartments, which are characterized by different progenitor subsets, spatial positioning and microenvironmental signals that jointly determine the outcomes of differentiation. On a larger scale, the activation of T cells leads to diverse parallel differentiation programs instead of one fixed one, stem-like, tissue-resident, and circulating T cell subsets are all distinct contributors to the immune response. In line with this, analysis at the single cell level showed that human CD8+ memory T cells contained functionally differentiated progenitors, with PD-1-TIGIT cells favoring functional lineages, and PD-1+TIGIT+ subsets adopting exhaustion-like condition (Galletti et al., 2020[72]). This initial diversification was also supported in the acute viral infection when individual CD8+ T cells combined antigenic and inflammatory signals by forming heterogeneous effector and memory cells via unique transcriptional regulons and enhancer landscapes (Schauder et al., 2021[215]). Simultaneously, cBAF and MYC were asymmetrically segregated in early activation, which added inherent heterogeneity, assigning daughter cells to an effector or a memory fate (Guo et al., 2022[87]). Similar to this, the Tfh cells with CD4+ were found to be descendant of fate, with TIGIT expression differentiating between germinal center and memory formation in the presence of c-Maf and Plekho1 (Zhu et al., 2023[290]).

T cell plasticity is also determined by localization of tissues and microenvironment, which dynamically determine functional specialization. In the peripheral tissues, intestinal TRM subsets was functionally heterogeneous with CD103+ maintains the resident ability with low recall capability and CD103- subsets maintained central memory-like characteristics and predominantly contributed to secondary responses (Fung et al., 2022[67]). The same was applied to tumor immunity with stem-like PD1+ TCF1+ CD4+ T cells becoming plastic with context-dependent changes in phenotype, adopting repressive iTreg cells in the presence of Tregs but adopting TH1 effector phenotypes with Treg depletion or TBET stimulation which enhanced tumor control by CD8+ T cells (Cardenas et al., 2024[25]). Furthermore, single-cell transcriptomic profiling of across human tissues showed that both the CD4+ and CD8+ T cells used site-selective transcriptional programmes, which reflects the presence of environmental signals and functional compartmentalization (Szabo et al., 2019[240]).

The formation of spatial organization of lymphoid tissues offers an extra mechanism of control that shapes the fate choice of T cells and maintains heterogeneity. More sophisticated imaging technologies showed that the differentiation of CD8+ T cells was spatially segregated in lymph nodes with CXCR3-based migration into interfollicular areas enhancing short-term formation of effector cells, whereas CCR7-based retention in the paracortex promoted the formation of stem-like memory cells (Duckworth et al., 2022[57]). Physical spatial programming was then enhanced by chemokine gradients in which T cells were directed by dendritic and stromal cell-secreted CXCL9 and CXCL10 to direct cell positioning, requesting an effector versus memory lineage commitment, and the stimulation of differentiation consequences by perturbing these cues (Duckworth et al., 2021[56]). All these results showed that T cell heterogeneity is both intrinsically and transcriptionally controlled and fundamentally relies on spatial location and microenvironmental structure, finally determining the equilibrium between effector activity and long-term immune memory.

## 7. Therapeutic Implications

### 7.1 Cancer immunotherapy

Successful cancer immunotherapy relies on controlled T cell activation, which can be influenced by checkpoint blockade, co-stimulation and overcoming immune-suppressive factors in the tumor microenvironment. A recent development has underscored the need for co-engagement of receptors for effective T cell activation. One study showed that the combination of antagonistic anti-PD-1 antibodies and agonistic anti-OX40 antibodies improved T cell activation, but only a small subset of T cells simultaneously received these signals when given as free antibodies. This challenge was addressed in dual immunotherapy nanoparticles that facilitated spatiotemporal co-delivery of anti-PD-1 and anti-OX40 antibodies. This significantly enhanced receptor co-engagement and signaling, leading to enhanced T cell activation in vitro and increased therapeutic responses in vivo, including enhanced immune memory (Mi et al., 2018[176]). Building on the importance of T cell-mediated tumor targeting, another study revealed that activated CD8⁺ T cells could induce ferroptosis in tumor cells by downregulating the cystine/glutamate antiporter system xc⁻, linking T cell activation directly to metabolic vulnerabilities in cancer cells. The combination of checkpoint blockade with cystine/cysteine depletion further amplified anti-tumor effects, demonstrating how T cell activation can be integrated with metabolic modulation to potentiate therapy (Wang et al., 2019[258]). Meanwhile, adenosine-mediated immunosuppression via A2A receptor was identified as a key inhibitory mechanism limiting T cell responses, with blockade of A2A receptor restoring CD8⁺ T cell function and reducing regulatory T cell accumulation, emphasizing the therapeutic potential of targeting inhibitory pathways alongside activation strategies (Vigano et al., 2019[252]). In addition, novel strategies aimed at restoring tumor-cell recognition and antigen-independent cytotoxicity have demonstrated promise in glioma models through modulation of USP14-PARP1 signaling pathways (Wang et al., 2026[257]). Alternative approaches, including bacteria-based tumor therapies and integrative immunotherapeutic strategies for glioblastoma, further expand the landscape of cancer immunotherapy (Lin et al., 2026[149]). Complementing these findings, studies on lymph nodes showed that progenitor-exhausted CD8⁺ T cells in uninvolved lymph nodes could differentiate into functional exhausted populations following PD-L1 blockade, highlighting the role of lymphoid tissue priming in systemic T cell-mediated anti-tumor immunity (Rahim et al., 2023[200]).

The dynamics of T cell activation have also been observed in clinical and preclinical tumor settings, revealing the impact of checkpoint inhibition on clonal expansion and systemic immune responses. In oral cancer patients, neoadjuvant anti-PD-1 or combined anti-PD-1/CTLA-4 therapy induced rapid expansion of tumor-infiltrating CD8⁺ T cells with enhanced cytotoxic and tissue-resident memory programs, showing that pre-existing activation states can determine responsiveness (Luoma et al., 2022[162]). Similarly, in glioblastoma, nanoparticle-mediated antigen delivery combined with localized hyperthermia promoted dendritic cell presentation and sustained T cell stimulation, illustrating how engineering approaches can overcome immune-privileged tumor barriers (Yalamandala et al., 2024[271]). Extending these strategies to adoptive therapies, a study targeting B7-H3 (CD276) in pediatric solid tumors demonstrated that CAR T cells could mediate robust antitumor activity, but efficacy depended on precise antigen density, emphasizing the necessity of coordinated T cell activation and tumor-specific targeting (Majzner et al., 2019[168]). In metastatic breast cancer, single-cell analyses revealed that alternative inhibitory interactions, such as LAG3-LGALS3 and TIGIT-NECTIN2, dominated over classical PD-1/PD-L1 pathways, suggesting that effective T cell activation may require targeting multiple suppressive axes to overcome complex immunosuppressive microenvironments (Zou et al., 2023[292]). A related study in gliomas uncovered a neuroimmune axis where neuron-derived midkine activated CD8⁺ T cells, which then stimulated microglia to support tumor stem cells, highlighting intricate intercellular mechanisms that influence T cell-mediated anti-tumor responses (Guo et al., 2020[90]).

Further refinements in co-stimulatory and combinatorial approaches have demonstrated improved T cell activation and therapeutic outcomes. Bispecific antibodies combining αTAA-αCD3 with co-stimulatory fusion proteins enhanced T cell proliferation, cytokine production, and cytotoxicity in solid tumor models, while overcoming immunosuppressive signaling from TGF-β and IL-10 (Warwas et al., 2021[260]). Tumor-derived factors such as progranulin were shown to suppress T cell activation via STAT3-mediated PD-L1 upregulation, whereas inhibition of this axis restored CD8⁺ T cell function, demonstrating the importance of counteracting tumor-mediated immunosuppression (Fang et al., 2021[63]). Engineering CAR T cells with cytokine co-expression (IL-15 and IL-21) and optimized costimulation improved proliferation, persistence, and antitumor efficacy in hepatocellular carcinoma models, illustrating how intrinsic T cell programming enhances therapeutic potential (Batra et al., 2020[13]). In combined approaches, activated Jurkat T cells synergized with miRNA-34a delivered via iron oxide nanorods to induce apoptosis and ferroptosis in lung cancer cells, highlighting how T cell activation can complement gene-based and nanotechnology therapies (Pandey et al., 2024[190]). Moreover, inhibition of DNA damage response proteins such as PARP and CHK1 increased PD-L1 expression and augmented CD8⁺ T cell infiltration, demonstrating that modulation of tumor-intrinsic pathways can further potentiate T cell-mediated immunotherapy (Sen et al., 2019[218]).

### 7.2 Autoimmune diseases

Dysregulated T cell responses are a fundamental mechanism in autoimmune diseases, in which unbalanced T effector cells cause tissue damage and defective regulatory T cells are unable to prevent immune responses. As such, therapeutic approaches specifically reshape T cell responses to achieve immune balance while avoiding global immunosuppression. This approach has informed strategies that exploit antigen specificity, T cell activation and inhibition, gene transcription, metabolism and tissue microenvironment to reshape aberrant T cell responses. One way to target T cell activation is through engineered antigen-specific T cells. In rheumatoid arthritis, universal FITC-based CAR-T cells targeted FITC-labeled citrullinated peptides to selectively deliver their cytotoxic effects to autoreactive B cells while leaving healthy B cells alone. The study showed that T cell activation could be targeted, in an antigen-specific way, to selectively kill pathologic cells (Zhang et al., 2021[282] ). Likewise in systemic lupus erythematosus (SLE), anti-CD19 CAR-T cells in mice led to persistent B cell depletion, disease prevention in pre-symptomatic animals, and superior disease control with 4-1BB co-stimulatory domains compared to CD28 co-stimulatory domains, highlighting the role of fine-tuning T cell co-stimulation in enhancing efficacy (Jin et al., 2021[113]). Other preclinical work also confirmed that CD8⁺ CAR-T cells selectively and persistently depleted autoreactive CD19⁺ B cells, which impeded autoantibody formation, alleviated organ-specific disease pathology and retained memory-like features, demonstrating that harnessing T cell activation can lead to long-term, antigen-specific immune suppression (Kansal et al., 2019[119]). In patients, treatment of refractory SLE, idiopathic inflammatory myositis, and systemic sclerosis with CD19-specific CAR-T cells induced disease remission, restored antibody levels, and enabled withdrawal of standard immunosuppressive drugs, suggesting clinical translation of this approach (Müller et al., 2024[182]).

Molecular-level modulation of T cell activation provides an alternate approach to engineered cellular therapies to reprogram aberrant immune responses. In SLE, silencing of microRNA-125a (miR-125a) promotes the activation of effector T cells and the suppression of regulatory T cells (Treg). Exposure to miR-125a nanoparticles delivered to splenic T cells restored the balance between effector and regulatory T cells, downregulating pathogenic T cell program, and promoting remission (Zhang et al., 2020[285]). In EAE, Lycium barbarum glycopeptide suppresses Th1/Th17 differentiation and promotes Treg development, suppressing neuroinflammation and demyelination via inhibition of the transcription factor AP-1, showing how local regulation of T cell activation can restore immune tolerance (Guo et al., 2026[88]). Alternatively, metabolic pathways were targeted: silencing of Pik3ip1 promoted aerobic glycolysis in T cells that worsened disease symptoms, while blockade of glycolysis or Hif1α restored metabolic balance, decreased hyperactivation of CD4⁺ T cells and resulted in amelioration of EAE (Xie et al., 2022[268]).

Returning to immune equilibrium in autoimmune diseases may involve boosting regulatory T cell activity or exploiting tissue-specific factors to regulate pathogenic T cells. In multiple sclerosis, brain-targeted delivery of IL-4 using helper-dependent adenoviral vectors facilitated homing of CD4⁺Foxp3⁺ Tregs into inflamed areas, restoring immune tolerance and enabling long-lasting clinical and neurophysiological improvement (Butti et al., 2008[22]). In inflammatory bowel disease (IBD), α1,3 fucosyltransferase VII (FUT7) was shown to be crucial for reintroducing Tregs to the gut; increasing FUT7 expression with nanocarriers promoted Treg homing to the gut, reduced disease severity, and improved immunosuppressive function (Liu et al., 2025[154]). Microbiome-derived metabolites like indole-3-propionic acid also regulate T cell activity by triggering apoptosis of pathogenic Th1 and Th17 cells, thereby alleviating inflammation in colitis (Gao et al., 2025[73]). Nanoparticle-based methotrexate delivery in collagen-induced arthritis induced selective suppression of CD4⁺IL-17⁺ T cells, while promoting activation of CD4⁺CD25⁺Foxp3⁺ Tregs, revealing co-ordinated control of adaptive immune subsets (Park et al., 2022[191]).

### 7.3 Infectious diseases

T cells play a crucial role in regulating infection, and the ability to appropriately activate or modify T cells can dictate the fate of previously intractable diseases. T cell-based strategies have offered both mechanistic insights and therapeutic benefits by boosting direct killing, directing helper cell subsets or reversing exhaustion. In fungal, bacterial and viral infections, approaches that exploit T cell function, such as adoptive transfer, receptor engineering, checkpoint mediation, or vaccination can re-establish immunity, limit the pathogen load and extend survival. In fungal infections, for instance, adoptively transferred chimeric antigen receptor T cells (Af-CAR T cells) specifically targeted the AB90-E8 antigen of Aspergillus fumigatus, allowing CD8+ T cells to lyse the fungus and CD4+/CD8+ T cells to release cytokines that activated macrophages. Adoptive transfer of T cells in immunodeficient mice targeted areas of infection, lowered fungal load, and enhanced survival (Seif et al., 2022[217]). Those results were confirmed by studies of cystic fibrosis patients, in whom chronic exposure to *A. fumigatus* resulted in long-term, antigen-specific activation of Th cells. Subsets of Th cells skewed towards Th1, Th2 or Th17 cells responded to different fungal proteins, and Th2-skewed responses were associated with allergic inflammation, demonstrating how the activation of different T cell subsets determines protective versus pathogenic responses (Schwarz et al., 2023[216]).

T cell exhaustion and immune checkpoints also determine infection outcomes but can be reversed. In invasive Candida albicans infections, T cells expressed PD-1 and TIGIT, leading to reduced cytokine production, which was reversed when the immune checkpoint blockade agent nivolumab was administered (Mellinghoff et al., 2022[174]). Similarly, in active tuberculosis, high PD-1/PD-L1 expression on CD4+ T cells and monocytes constrained immune cell proliferation and the ability of macrophages to kill Mycobacterium tuberculosis, but blockade of this pathway in vitro restored T cell expansion and innate immune responses (Shen et al., 2016[222]). Moving from mechanisms to treatment, repeated infusion of allogeneic Vγ9Vδ2 T cells in patients with multidrug-resistant tuberculosis improved immune cell activity, promoted repair of lung lesions, and improved clinical outcome by boosting the killing of M. tuberculosis, linking mechanistic insights into T cell regulation with therapeutic outcomes (Liang et al., 2021[146]).

Engineering and vaccination approaches also extend these concepts to viruses and viral reservoirs. TCR engineering via circular mRNA (cmRNA) allowed sustained expression of antigen-specific TCRs that recognized CMV pp65 and facilitated T cell growth and killing of infected cells in vitro and in vivo. In clinical applications, CMV-specific T cells were temporarily lost following CAR T cell treatment but could be tracked and augmented with cmRNA-TCR-Ts to restore efficient antiviral responses, bridging mechanistic design principles with clinical outcomes (Kampouri et al., 2023[118]). Likewise, CAR T cells that bind HIV-infected cells in an MHC-independent manner persisted and remained highly cytotoxic (York et al., 2022[278]). Additionally, broadly neutralizing antibody CAR T cells reduced viral RNA and intact proviruses in ART-treated patients, creating evolutionary pressure on the virus and demonstrating T cell activation can target the latent reservoir (Liu et al., 2021[152]). SARS-CoV-2 mRNA vaccines also generated IFN-γ-secreting CD4+ and CD8+ T cells, even in immunocompromised patients, demonstrating the ability of T cell activation to drive protective immunity (Tortorella et al., 2022[243]). Further, preexisting T cell responses with the ability to kill and modulate regulatory responses to SpCas9 from *Streptococcus pyogenes* highlighted the therapeutic potential and safety implications of T cells in this new frontier of therapies (Wagner et al., 2019[253]).

## 8. Current Challenges and Controversies

An open question, and a continuing controversial issue in T cell biology is the gap between simplified experimental approaches and physiologically relevant systems. Traditional in vitro methods such as plate-bound anti-CD3/28 stimulation, or PMA/ionomycin stimulation, cannot recapitulate events which occur at the antigen present cell (APC)-T cell contact, such as spatial distribution, diversity and mechanical forces arising at the synapse. Thus, signaling events observed in such systems may be quite distinct to those observed in vivo and raise questions of ecological validly and overinterpretation of basic outcomes (Depoil and Dustin, 2014[51]; Courtney et al., 2018[42]). This lack of connection is a major cause of the problem of reproducibility, where experimental results from highly controlled in vitro experiments don't always repeat across laboratories and time, or translate to in vivo systems, particularly in studies focusing on co-stimulation, exhaustion and memory differentiation (Gaud et al., 2018[76]). Single-cell technology is embracing, and introduces, more complexity and controversy. While single cell RNA sequencing (scRNA-seq) and other single cell multi-omics technologies have revealed dramatic diversity in T cell states, these data may not always be straightforward to interpret. It is difficult to account for the technical and biological variation between important cell states and the labile activation signatures or computational artefacts and computational pipelines may yield different answers depending on the methodology used to calculate the cell states (Stuart and Satija, 2019[238]; Lähnemann et al., 2020[126]). Furthermore, the gene expression profile is sometimes not helpful in being a direct readout of the function of T cell, as protein modification, location and dynamics of T cell signaling are crucial in T cell function. These restrictions are also amplified in the translational blocks, in which results obtained using animal models and/or ex vivo human studies are generally not predictive of clinical responses in immunotherapy. Heterogeneity of tumor microenvironment, patient to patient variability and immune history all hampers the translation of mechanistic findings into therapeutic responses (Sharma et al., 2021[221]). All these issues underline the need for an integrated and contextualized approach to achieve a balance between experimental precision and physiological relevance.

Another big issue is the lack of standardized frameworks for benchmarking experimental T cell activation models. The biology of immune cells differs in many ways between different models of antibody activation, including plate-based activation, artificial antigen-presenting cells, organoid cultures, microfluidic systems, and in vivo models. Thus, a variation between studies may not actually be experimental error, but rather biological variation as imposed by the model. Normalization of reporting standards, reference datasets, standardized analytical pipelines, and orthogonal validation strategies, will hence be critical to increase the reproducibility across laboratories. Importantly, future work should focus on experiments that are physiologically relevant and at the same time are experimentally tractable, such that fundamental mechanistic findings can be translated to experiments in human disease.

## 9. Future Directions

New transformative technologies to introduce new analytical capabilities, such as interrogation of immune responses in their native spatial and molecular environment, are defining the next phase of T cell research. The recent method of spatial transcriptomics and in situ proteomics enable expression and protein distribution mapping of the intact tissues simultaneously and maintains important data of cell-cell interactions and microenvironmental stimuli. Such methods are especially effective in breaking down tumor-immunomodulatory interfaces where the geometrical positioning of the T cells verses the antigen presentation cells, stromal barriers, and utter niches determine functional responses (Mund et al., 2022[183]). Simultaneously, artificial intelligence (AI)-based modeling is becoming one of the primary tools related to the combination of high-dimensional data, discovery of latent patterns, and predicting the signaling results. Machine learning models are being used to decode TCR specificity to androgen antigens, and recapitulate signaling networks, as well as stratifying patients to receive immunotherapy, but matters of interpretability and data bias continue to be of concern (Greener et al., 2022[85]).

One conceptual transformation in the field is to shift to a systems level comprehension of T cell activation that encompasses processes at many scales, between molecular signaling events and metabolic states and cellular interactions as well as tissue-scale organization. Multi-scale modeling frameworks where experimental data and assertive simulations coexist are starting to cross these layers providing information about the expansion of the local signaling dynamic to encompass population-level behaviors and immune outcomes (Malissen et al., 2014[169]; Davis et al., 2017[47]). These developments are eventually moving towards the state of predictive immunology, in which T cell reaction can be quantitatively simulated and engineered in a patient-specific form. These methodologies promise the sensible evolution of customized immunotherapies, such as the ready tiered agencies blockade plans, custom-built T cell therapies, and love made vaccine platforms. Nevertheless, this vision is going to need strict standardization, cross-disciplinary teamwork, and extrapolation of longitudinal clinical data to make sure that predictive models are robust and can be acted upon in a clinical setting.

An important future challenge is to build integrated model selection frameworks that can facilitate selecting the most suitable experimental platform to meet specific biological questions. Moving forward, in vitro, ex vivo, organoid, animal, and computational systems should not be thought of as mutually exclusive systems, but rather complementary tools as part of a translational continuum. Such a scheme would enable rational choice of experimental design, reduce the variation between experiments, and the transfer of new discoveries into clinically relevant treatments with immunotherapeutic agents.

## 10. Conclusion

T cell activation is an exquisitely orchestrated and complex process. So, T cell activation should not be viewed as an enzymatic pathway but as a dynamic systems-level network that links events at the immunological synapse and at the receptor proximal sites to downstream transcriptional, metabolic, and functional events. The ensemble network of known signal pathways, including calcium-NFAT, MAPK, NF-kB and PI3K-Akt-mTOR, enables digital and analog processing of thresholds and tuning of the activation process and control for sensitivity, specificity and tolerance. Importantly, advances in imaging, single cell multi-omics approaches and mechanobiology have changed the way we look at how T cells respond to the environment. These approaches highlight that the processes involved in activation are controlled not only by ligand-receptor affinity or contact with a receptor, but also by mechanics, prearrangement and temporal assembly of signals. These advances challenge the reductionist approaches to T cell activation, and highlight the incapability of often used in vitro based models to effectively represent circumstellar physiological antigen presentation and cell-cell interactions. The cumulative view of these two aspects, through a translational lens, is impacting drug design, cancer immunotherapy, vaccine, autoimmune disease and infectious disease. Increasingly, the paradigm of single pathway- or receptor-specific intervention in therapy is deemed insufficient owing to the complexity of T cell signaling network redundancy and cross-regulation. Instead, therapies should consider the cellular signaling as an additive and redundant event, such as immune checkpoint blockade therapy and next-generation engineered CAR-T cell therapy is a gourmet meal to co-stimulatory and metabolic pathways.

A prevailing theme that comes from this review is that, as research in the T cell field continues to discover new aspects of the mechanisms underlying these processes, it also becomes more and more essential to choose physiologically relevant experimental systems. Together, comparative evaluation of activation models, integration of multi-omics datasets, and use of systems level computational approaches offer a framework for connecting the dots between mechanistic discovery and clinical translation. Future studies that focus on methodological rigor, reproducibility, and applicability to clinical translation will be better able to provide comprehensive and clinically significant T cell insights. Progress in the future will not be possible through single-tackled or full-arm approaches, but by the significance of such a combination in capturing the full complexity of T cell biology.

## Declaration

### Author contributions

Mohammed Ageeli Hakami: Data Curation, Investigation, Writing - Original Draft. Rabab Fatima: Investigation, Methodology, Writing - Original Draft. Yumna Khan: Conceptualization, Data Curation, Software, Visualization. Ahad Amer Alsaiari: Conceptualization, Investigation, Writing - Original Draft. Jawaher Amer Alsaiari: Formal Analysis, Visualization, Writing - Review & Editing. Md Sadique Hussain: Conceptualization, Supervision, Writing - Review & Editing, Project Administration.

All authors have approved the final version of the manuscript.

### Artificial Intelligence (AI) - assisted technology

During the preparation of this manuscript, the authors used ChatGPT solely for the purpose of improving grammar, spelling, and language clarity. The AI tool was not used to generate scientific content, data interpretation, or conclusions. All outputs were carefully reviewed and edited by the authors, who take full responsibility for the accuracy, integrity, and originality of the final manuscript.

### Conflict of interest

Declared none.

### Acknowledgments

The authors would like to thank the Deanship of Scientific Research at Shaqra University for supporting this work.

### Funding

None.

### Clinical trial number

Not applicable.

### Data availability

Not applicable.

### Ethics approval

Not applicable.

### Informed consent

Not applicable.

## Figures and Tables

**Table 1:**
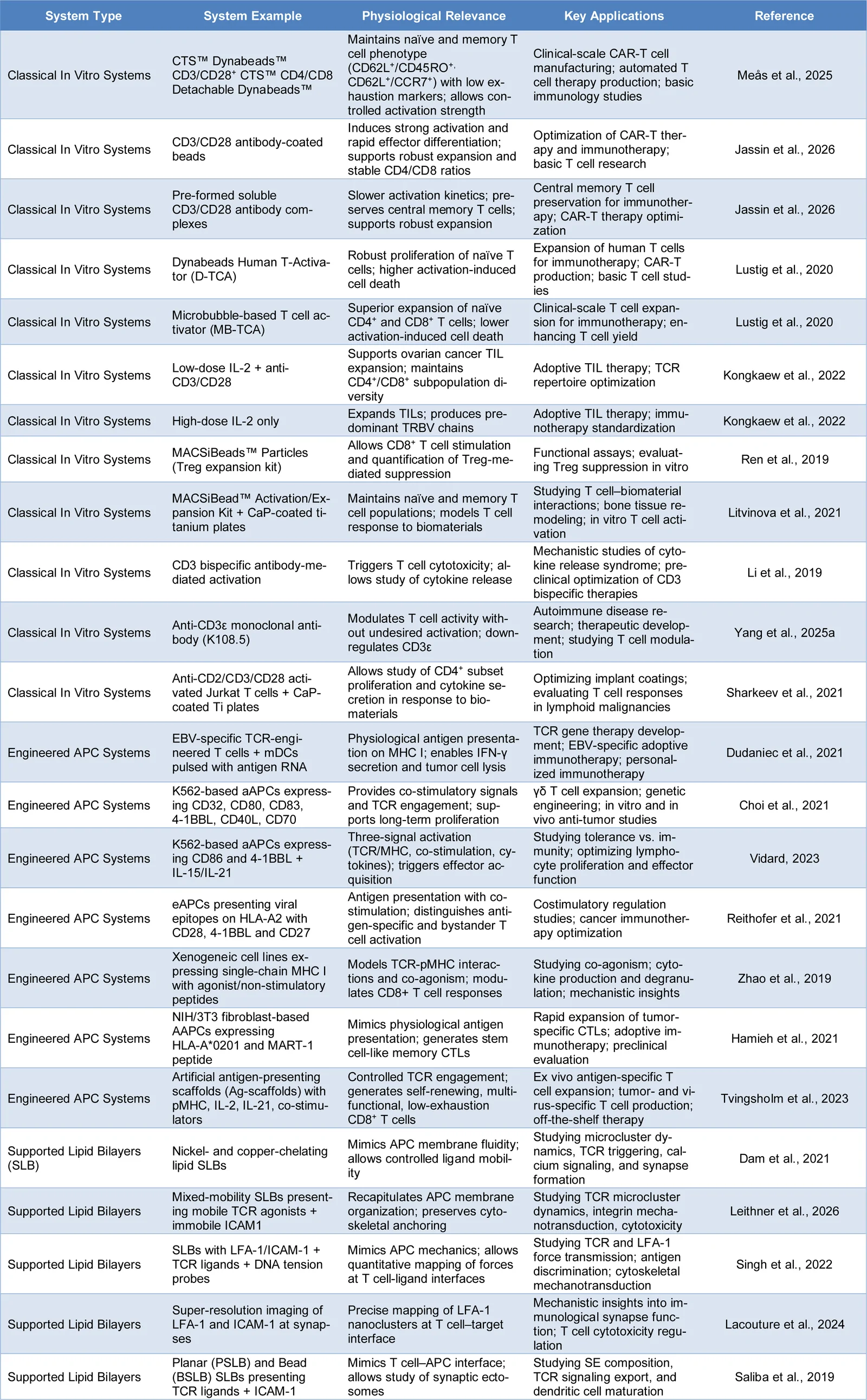
Experimental models of T cell activation: Physiological relevance and applications

**Figure 1:**
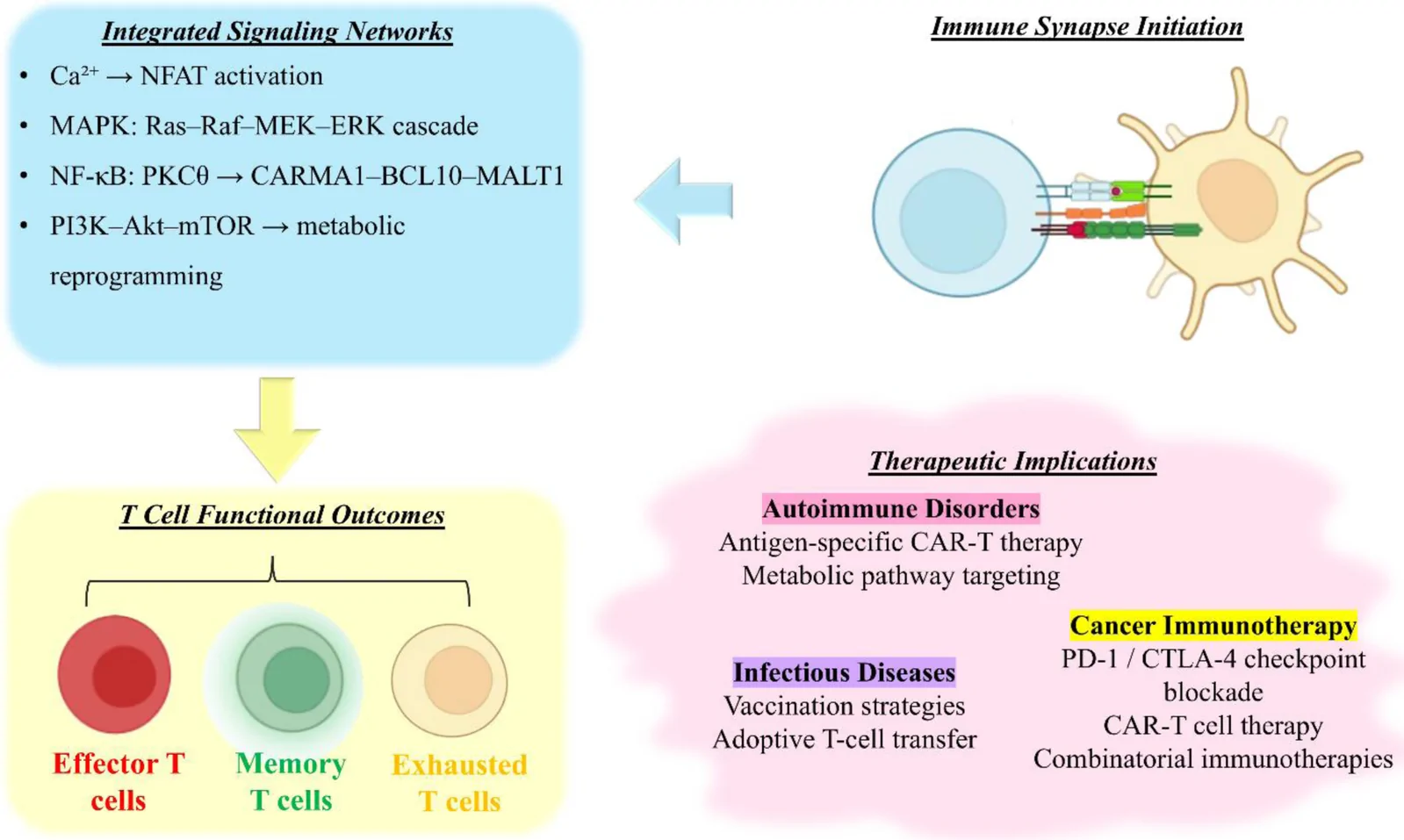
Graphical abstract
